# Use of non-steroidal anti-inflammatory drugs and risk of death from COVID-19: an OpenSAFELY cohort analysis based on two cohorts

**DOI:** 10.1136/annrheumdis-2020-219517

**Published:** 2021-01-21

**Authors:** Angel YS Wong, Brian MacKenna, Caroline E Morton, Anna Schultze, Alex J Walker, Krishnan Bhaskaran, Jeremy P Brown, Christopher T Rentsch, Elizabeth Williamson, Henry Drysdale, Richard Croker, Seb Bacon, William Hulme, Chris Bates, Helen J Curtis, Amir Mehrkar, David Evans, Peter Inglesby, Jonathan Cockburn, Helen I McDonald, Laurie Tomlinson, Rohini Mathur, Kevin Wing, Harriet Forbes, Rosalind M Eggo, John Parry, Frank Hester, Sam Harper, Stephen JW Evans, Liam Smeeth, Ian J Douglas, Ben Goldacre, The OpenSAFELY Collaborative

**Affiliations:** 1 Department of Non-Communicable Disease Epidemiology, London School of Hygiene & Tropical Medicine, London, UK; 2 The DataLab, Nuffield Department of Primary Care Health Sciences, University of Oxford, Oxford, Oxfordshire, UK; 3 TPP, Leeds, UK

**Keywords:** arthritis, rheumatoid, COVID-19, epidemiology, osteoarthritis

## Abstract

**Objectives:**

To assess the association between routinely prescribed non-steroidal anti-inflammatory drugs (NSAIDs) and deaths from COVID-19 using OpenSAFELY, a secure analytical platform.

**Methods:**

We conducted two cohort studies from 1 March to 14 June 2020. Working on behalf of National Health Service England, we used routine clinical data in England linked to death data. In study 1, we identified people with an NSAID prescription in the last 3 years from the general population. In study 2, we identified people with rheumatoid arthritis/osteoarthritis. We defined exposure as current NSAID prescription within the 4 months before 1 March 2020. We used Cox regression to estimate HRs for COVID-19 related death in people currently prescribed NSAIDs, compared with those not currently prescribed NSAIDs, accounting for age, sex, comorbidities, other medications and geographical region.

**Results:**

In study 1, we included 536 423 current NSAID users and 1 927 284 non-users in the general population. We observed no evidence of difference in risk of COVID-19 related death associated with current use (HR 0.96, 95% CI 0.80 to 1.14) in the multivariable-adjusted model. In study 2, we included 1 708 781 people with rheumatoid arthritis/osteoarthritis, of whom 175 495 (10%) were current NSAID users. In the multivariable-adjusted model, we observed a lower risk of COVID-19 related death (HR 0.78, 95% CI 0.64 to 0.94) associated with current use of NSAID versus non-use.

**Conclusions:**

We found no evidence of a harmful effect of routinely prescribed NSAIDs on COVID-19 related deaths. Risks of COVID-19 do not need to influence decisions about the routine therapeutic use of NSAIDs.

**Key messages**

**What is already known about this subject?**

- There have been concerns that non-steroidal anti-inflammatory drugs (NSAIDs) may increase the risk of COVID-19 disease. Recent observational studies reported no evidence of a harmful effect of NSAID use on COVID-19 severity among patients with COVID-19.
- However, most studies were of much smaller sample size, not general population based or did not specifically investigate individual NSAIDs (eg, naproxen and ibuprofen).
- In addition, limited clinical data are available to advise patients using long-term NSAID treatment (including people with rheumatoid arthritis and osteoarthritis) whether the treatment should be continued or stopped in the context of COVID-19 pandemic.

**What does this study add?**

- We identified two study populations (2 463 707 people who ever used NSAIDs in the past 3 years from the general population and 1 708 781 people with rheumatoid arthritis/osteoarthritis) in England using OpenSAFELY platform. We then grouped them into current users and non-users, respectively, in each study population.
- In both populations, no association between NSAIDs and COVID-19 related death was found.

**How might this impact on clinical practice or future developments?**

- This study does not support the hypothesis of any harmful effect of NSAIDs on COVID-19 related deaths among regular NSAID users.
- Treatment decisions about the routine use of NSAIDs do not need to be influenced by fears of an effect on COVID-19 outcomes.

## Introduction

COVID-19, caused by the SARS-CoV-2, has been diagnosed in approximately 18 million patients with >690 000 deaths in >200 countries as of 5 August 2020.^1^

Non-steroidal anti-inflammatory drugs (NSAIDs) are widely prescribed for relief of pain and inflammation with nearly 11 million NSAID prescriptions dispensed in primary care in England in the last 12 months.^2^ Additionally, some NSAIDs (eg, ibuprofen and aspirin) are available for sale without a prescription with a single brand of ibuprofen alone having sales of approximately £100 million per annum.^3^ Nine non-interventional studies have suggested that NSAIDs may be associated with increased risk of complications of lower respiratory tract infections^4–12^; though there is evidence that indometacin may have protective antiviral effects reported from a single animal study.^13^

There is now a debate over whether NSAIDs may worsen the prognosis of COVID-19. On 14 March, it was recommended in France that patients should avoid NSAID use due to an apparent worsening of COVID-19 in those taking ibuprofen, based on unpublished reports.^14^ This gained worldwide attention and resulted in the National Health Service (NHS) England medical director issuing a directive that paracetamol should be used in preference to NSAIDs^14^ for symptoms of COVID-19. Subsequent reviews by USA, UK and EU drug regulators^15–17^ recommended that individuals currently using NSAIDs for the management of chronic diseases should continue the treatment while calling for more evidence of the impact of NSAIDs in patients with COVID-19. Two systematic reviews highlighted a lack of studies investigating the effect of NSAIDs on COVID-19, demonstrating the urgent need of new studies.^18 19^ One cohort study was recently conducted to investigate such association, but individual NSAIDs were not specifically investigated.^20^

We therefore investigated the association between NSAID use and deaths from COVID-19 using linked data from >17 million patients in England. We further examined whether the association varied by types of NSAID.

## Methods

### Study design

We conducted two cohort studies using primary care electronic health record data linked to death data from the Office for National Statistics between 1 March 2020 and 14 June 2020.

### Data source

Primary care records managed by the software provider The Phoenix Partnership (TPP) were linked to Office for National Statistics death data through OpenSAFELY, a data analytics platform created by our team on behalf of NHS England.^21^ The dataset analysed within OpenSAFELY is based on 24 million people currently registered with primary care practices using The Phoenix Partnership SystmOne software, representing 40% of the English population. It includes pseudonymised data such as coded diagnoses, prescribed medications and physiological parameters.

### Study populations

We identified two cohorts, anticipating that underlying factors influencing NSAID use and therefore potential biases would differ between them. The first cohort was all people with ≥1 oral NSAID prescription within the 3 years before study start (1 March 2020), identified from the general population. It was chosen to minimise confounding by restricting to people who were currently prescribed NSAIDs and those who recently stopped NSAIDs as their characteristics were likely to be more comparable than never-users. The second cohort was all people with a diagnosis of rheumatoid arthritis (RA)/osteoarthritis (OA) before study start. It was chosen because they were potential NSAID users with similar underlying diseases to reduce confounding by indication. A patient could be included in both cohorts.

In both cohorts, people with missing data for gender, index of multiple deprivation, <1 year of primary care records or aged <18 or >110 years were excluded. Aspirin is used at lower doses as an antiplatelet to prevent cardiovascular disease,^22^ indicating aspirin users constitute a different population from other NSAID users. We therefore excluded people ever prescribed aspirin in the 10 years before study start or a record of either stroke or myocardial infarction before study start. We excluded people with a record of gastrointestinal bleeding or current asthma before the study start, as they are contraindications to NSAIDs.^22^

### Exposures

In the main analysis, we defined current NSAID users as those ever prescribed NSAID in the 4 months prior to study start, and non-users are those with no record of NSAID prescription in the same time period.

We examined whether the association varied by types of NSAID, specifically: (1) naproxen dose (categorised as non-use, high-dose naproxen (500 mg), low-dose naproxen (250 mg) and other NSAIDs based on the strength of the formulation), (2) COX-2 specific NSAIDs (categorised as non-use, COX-2 specific (celecoxib/etoricoxib) and non-specific NSAIDs) and (3) ibuprofen (categorised as non-use, ibuprofen and other NSAIDs).

### Outcomes

Follow-up for each cohort began on the 1 March 2020 and ended either on date of death or study end date (14 June 2020). If people in the non-user group received a NSAID prescription after 1 March 2020, they were censored at the date of this prescription (online supplemental figure S1).

10.1136/annrheumdis-2020-219517.supp1 Supplementary data

The outcome was COVID-19 related death as registered in Office for National Statistics data using International Classification of Diseases (ICD)-10 codes U07.1 (‘COVID-19, virus identified’) and U07.2 (‘COVID-19, virus not identified’) listed either as the underlying or any contributing cause of death. The latter ICD-10 code is used when laboratory testing is inconclusive or unavailable.^23^

### Covariates

**Figure 1:**
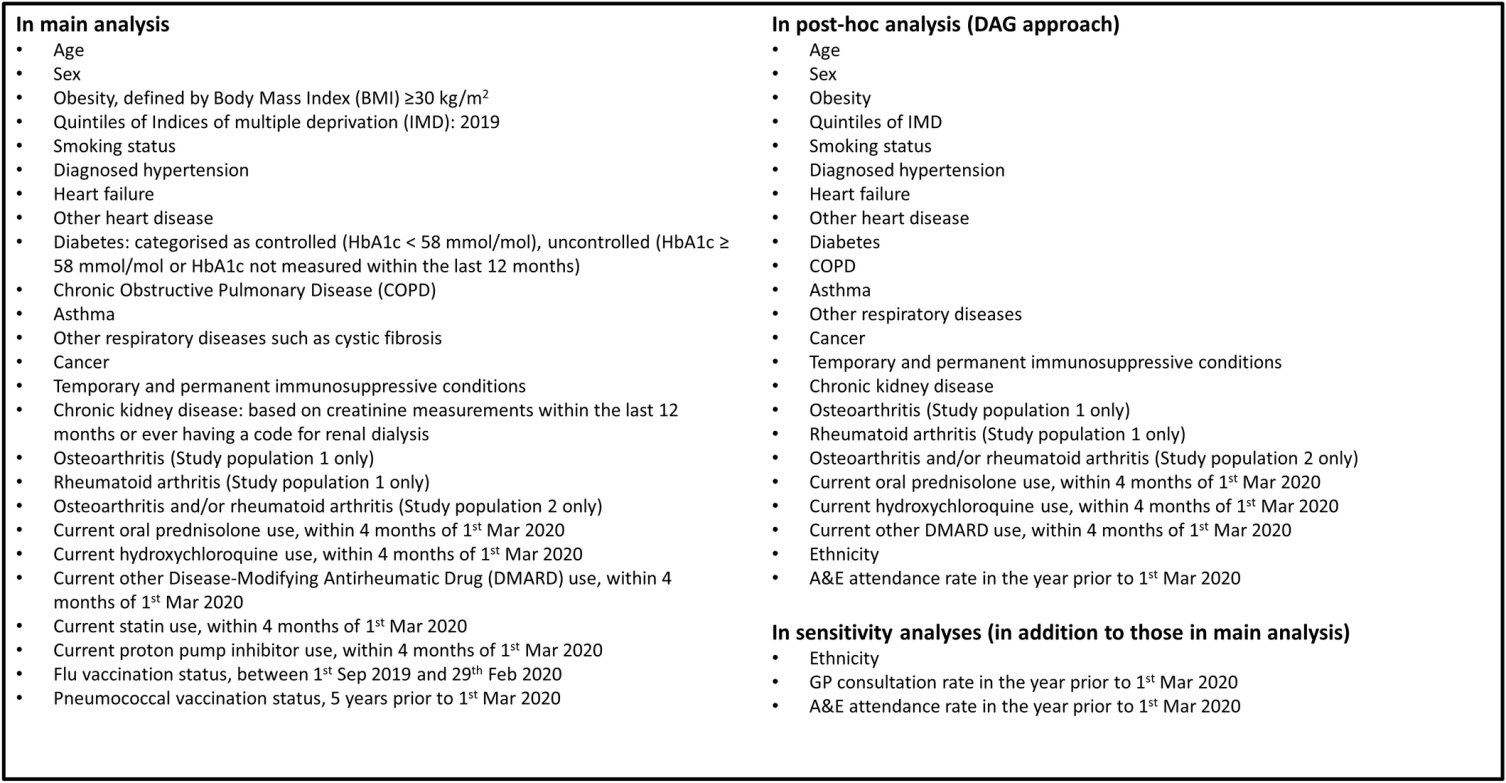
Prespecified hypothetical confounders. A&E, accident & emergency; DMARD, disease-modifying antirheumatic drugs; HbA1c, hemoglobin A1c; GP, general practice.

Figure 1 presents the final list of potential confounders. Our methodology for creating codelists for variables has been previously described.^21^ All codelists for identifying exposures, covariates and outcomes are openly shared at https://codelists.opensafely.org/.

### Statistical methods

Baseline characteristics in each cohort were summarised using descriptive statistics, stratified by exposure status. Time to COVID-19 related death was displayed in Kaplan-Meier plots. We present adjusted cumulative mortality curves and the difference between curves using the Royston-Parmar model. We estimated HRs with 95% CIs for the association between current NSAID use and COVID-19 related death using Cox regression with time since cohort entry as the underlying timescale. We accounted for competing risk by modelling the cause-specific hazard (ie, censoring non-COVID-19 deaths). We used graphical methods and tests based on Schoenfeld residuals to explore violations of the proportional hazards assumption.

Unadjusted models, models adjusted for age (using restricted cubic splines) and sex and multivariable-adjusted models including covariates listed in figure 1 were fitted. We stratified the multivariable-adjusted models by geographical regions, defined by Sustainability and Transformation Partnerships,^24^ to account for between-region variations. We evaluated the variation by age (under and 70+ years old) and performed likelihood ratio tests to analyse effect modification.

### Quantitative bias analysis

We used e-value formulae to calculate the minimum necessary strengths of association between an unmeasured confounder and exposure or outcome, conditional on measured covariates, to fully explain observed non-null adjusted associations (ie, to move the observed non-null association to the null).^25^

### Sensitivity analyses

Table 1 shows the list of sensitivity analyses.

**Table 1:** List of sensitivity analyses

Sensitivity analysis	Justification
1. Additionally adjusted for ethnicity in multivariable-adjusted models.	In the main analysis, we did not adjust for ethnicity as it was not anticipated to be a strong confounder and due to a sizeable proportion of individuals with missing ethnicity (~23%). We undertook complete case analysis to address missing data.
2. Additionally adjusted for the number of primary care consultations and A&E attendance in the past year in multivariable-adjusted models.	To explore the impact of healthcare-seeking behaviours.
3. For covariate of diabetes severity, we separated people with diabetes diagnosis and HbA1c measures ≥58 mmol/mol and those with diabetes diagnosis but without HbA1c measures in the past year into two different categories.	People with a diabetes diagnosis but not having HbA1c measures in the past year are likely to have uncontrolled diabetes due to their potential lack of monitoring and management of diabetes. Therefore, we classified these people as uncontrolled diabetes in the main analysis. This is an analysis to test the sensitivity of the results.
4. Repeated main analysis with a choice of covariates selected by a DAG approach (post hoc analysis).	To test the robustness of the results by choosing a set of covariates that are confounders with the use of a structured visual presentation (online supplemental figure S2).
5. Repeated main analysis varying the definition of currently prescribed an NSAID to within 2 months of 1 March 2020.	To assess the sensitivity of exposure definition.
6. Repeated main analysis excluding indometacin from all NSAIDs as the exposure of interest.	Indometacin was the only NSAID that was suggested to have antiviral activity against SARS virus.^13^
7. Repeated main analysis without censoring people who were prescribed NSAIDs after study start date in the non-use group.	To examine data as an intention-to-treat analysis, in order to limit potential bias due to informative censoring.
8. Repeated main analysis excluding people ever prescribed aspirin before study start date.	To assess the sensitivity of exclusion criteria.

DAG, directed acyclic graph; NSAID, non-steroidal anti-inflammatory drug.

### Software and reproducibility

Data management was performed using Python V.3.8 and SQL, with analysis carried out using Stata V.16.1. All study analyses were preplanned unless otherwise stated. All code for data management and analyses in addition to the prespecified protocol are archived at: https://github.com/opensafely/nsaids-covid-research.

### Patient and public involvement

Patients were not formally involved in developing this specific study design that was developed rapidly in the context of a global health emergency. We have developed a publicly available website https://opensafely.org/ through which we invite any patient or member of the public to contact us regarding this study.

## Results

Online supplemental figure S3 shows the flow chart of inclusion of participants. A total of 561 027 (13%) individuals were included in both study populations. Of them, 175 495 (25%) were current NSAID users and 385 532 (11%) were non-users.

## Main analysis

### Study population 1: general population

#### Patient characteristics

We included 536 423 current NSAID users and 1 927 284 non-users (table 2). Median age was 53 years (IQR 42–64) among current users and 49 years (IQR 36–60) among non-users. More women were current users (59.2%) than non-users (56.7%).

**Table 2:** Demographic and clinical characteristics

	Study population 1: general population (people prescribed NSAIDs in the past 3 years)		Study population 2: patients with rheumatoid arthritis or osteoarthritis	
	Non-use of NSAIDs	Current use of NSAIDS	Non-use of NSAIDs	Current use of NSAIDS
Total	**1 927 284**	**536 423**	**1 533 286**	**175 495**
Age as of 1 March 2020				
18–<40	598 513 (31.1)	115 858 (21.6)	32 958 (2.1)	4433 (2.5)
40–<50	397 201 (20.6)	103 076 (19.2)	97 870 (6.4)	15 813 (9.0)
50–<60	423 937 (22.0)	133 066 (24.8)	292 186 (19.1)	45 397 (25.9)
60–<70	283 639 (14.7)	106 205 (19.8)	416 489 (27.2)	56 947 (32.4)
70–<80	169 281 (8.8)	62 221 (11.6)	436 477 (28.5)	41 350 (23.6)
80+	54 713 (2.8)	15 997 (3.0)	257 306 (16.8)	11 555 (6.6)
Median, IQR	49 (36–60)	53 (42–64)	68 (58–76)	63 (55–71)
Sex				
Female	1 093 581 (56.7)	317 341 (59.2)	951 417 (62.1)	110 526 (63.0)
Body mass index				
<18.5	26 435 (1.4)	6041 (1.1)	19 616 (1.3)	1260 (0.7)
18.5–24.9	484 862 (25.2)	114 657 (21.4)	379 233 (24.7)	31 531 (18.0)
25–29.9	577 087 (29.9)	159 573 (29.7)	518 602 (33.8)	55 387 (31.6)
30–34.9	333 254 (17.3)	106 314 (19.8)	298 505 (19.5)	40 513 (23.1)
35–39.9	138 059 (7.2)	50 406 (9.4)	119 286 (7.8)	20 062 (11.4)
40+	71 503 (3.7)	30 438 (5.7)	58 801 (3.8)	12 396 (7.1)
Missing	296 084 (15.4)	68 994 (12.9)	139 243 (9.1)	14 346 (8.2)
Ethnicity				
White	1 236 854 (64.2)	357 651 (66.7)	1 095 982 (71.5)	125 073 (71.3)
Mixed	20 556 (1.1)	4696 (0.9)	6563 (0.4)	830 (0.5)
Asian/Asian British	151 533 (7.9)	33 010 (6.2)	51 587 (3.4)	6969 (4.0)
Black	49 618 (2.6)	10 527 (2.0)	17 645 (1.2)	2106 (1.2)
Other	30 214 (1.6)	6925 (1.3)	10 916 (0.7)	1241 (0.7)
Missing	438 509 (22.8)	123 614 (23.0)	350 593 (22.9)	39 276 (22.4)
Index of multiple deprivation				
1 (least deprived)	388 369 (20.2)	107 541 (20.0)	313 701 (20.5)	30 797 (17.5)
2	387 428 (20.1)	108 997 (20.3)	309 372 (20.2)	32 946 (18.8)
3	382 357 (19.8)	107 626 (20.1)	307 669 (20.1)	34 597 (19.7)
4	384 598 (20.0)	106 598 (19.9)	303 859 (19.8)	36 682 (20.9)
5 (most deprived)	384 532 (20.0)	105 661 (19.7)	298 685 (19.5)	40 473 (23.1)
Smoking status				
Never	841 256 (43.6)	220 293 (41.1)	672 833 (43.9)	70 283 (40.0)
Former	665 068 (34.5)	207 354 (38.7)	692 164 (45.1)	80 983 (46.1)
Current	389 340 (20.2)	103 258 (19.2)	164 464 (10.7)	23 913 (13.6)
Missing	31 620 (1.6)	5518 (1.0)	3825 (0.2)	316 (0.2)
Comorbidities				
Hypertension	353 803 (18.4)	128 078 (23.9)	625 247 (40.8)	66 098 (37.7)
Heart failure	9512 (0.5)	2433 (0.5)	36 888 (2.4)	1413 (0.8)
Other heart disease	27 881 (1.4)	8726 (1.6)	57 976 (3.8)	4202 (2.4)
Diabetes				
Controlled (HbA1c <58 mmol/mol)	122 653 (6.4)	42 132 (7.9)	177 397 (11.6)	19 535 (11.1)
Uncontrolled (HbA1c ≥58 mmol/mol)	50 268 (2.6)	16 504 (3.1)	58 452 (3.8)	6286 (3.6)
HbA1c not measured	4536 (0.2)	1303 (0.2)	3695 (0.2)	419 (0.2)
COPD	42 636 (2.2)	15 435 (2.9)	85 858 (5.6)	8373 (4.8)
Other respiratory diseases	17 270 (0.9)	6194 (1.2)	38 248 (2.5)	3435 (2.0)
Cancer	95 315 (4.9)	32 128 (6.0)	174 647 (11.4)	15 940 (9.1)
Immunosuppression	9285 (0.5)	2918 (0.5)	8498 (0.6)	1009 (0.6)
Chronic kidney disease	51 642 (2.7)	17 570 (3.3)	164 985 (10.8)	11 148 (6.4)
Osteoarthritis	367 954 (19.1)	162 676 (30.3)	1 473 833 (96.1)	162 676 (92.7)
Rheumatoid arthritis	28 690 (1.5)	21 526 (4.0)	95 056 (6.2)	21 526 (12.3)
Primary care consultations				
Median, IQR	5 (2–10)	7 (4–13)	6 (3–11)	8 (5–14)
Min, Max	0, 626	0, 576	0, 468	0, 360
A&E attendance				
Median, IQR	0 (0–0)	0 (0–0)	0 (0–0)	0 (0–0)
Min, Max	0, 118	0, 152	0, 71	0, 63
Vaccination				
Influenza	435 383 (22.6)	162 082 (30.2)	806 064 (52.6)	86 515 (49.3)
Pneumococcal	116 464 (6.0)	44 902 (8.4)	193 145 (12.6)	24 744 (14.1)
Medications				
Statin	223 221 (11.6)	87 169 (16.3)	415 120 (27.1)	47 020 (26.8)
Proton-pump inhibitors	268 934 (14.0)	342 266 (63.8)	371 464 (24.2)	137 180 (78.2)
Oral prednisolone	39 081 (2.0)	16 084 (3.0)	61 256 (4.0)	8265 (4.7)
Hydroxychloroquine	8074 (0.4)	6680 (1.2)	16 783 (1.1)	5104 (2.9)
Other DMARDs	20 770 (1.1)	16 857 (3.1)	48 819 (3.2)	12 753 (7.3)

A&E, accident & emergency; COPD, chronic obstructive pulmonary disease; DMARDs, disease-modifying antirheumatic drugs; HbA1c, hemoglobin A1c; NSAIDs, non-steroidal anti-inflammatory drugs.

Current users were more likely to be obese, former smokers and have a medical history of hypertension, diabetes, other respiratory diseases, cancer, chronic kidney disease, OA and RA than non-users. Current users were also more likely to have a prescription for statins, proton pump inhibitors and disease-modifying antirheumatic drugs and to have had more primary care consultations and vaccinations than non-users.

#### Unadjusted and multivariable results

**Figure 2:**
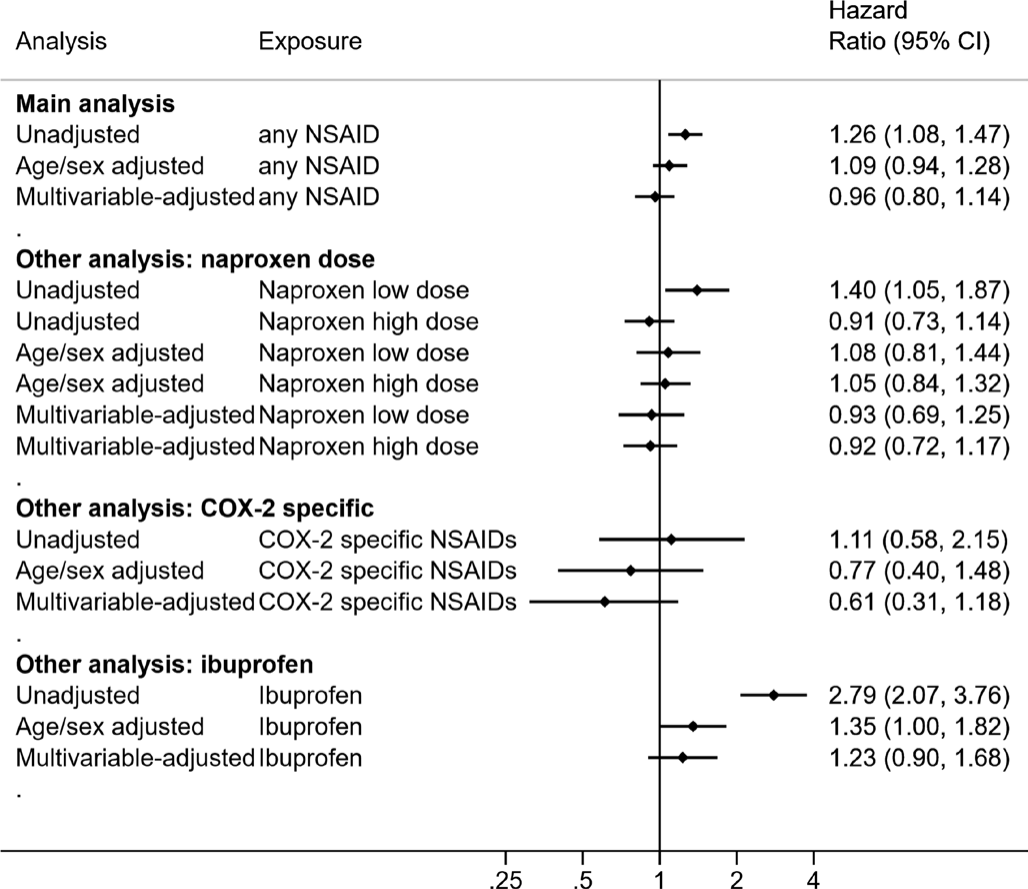
HRs of the association between current use of NSAIDs and COVID-19 related deaths in the general population. NSAIDs, non-steroidal anti-inflammatory drugs.

Online supplemental figures S4 and S5 present time to COVID-19 related death in Kaplan-Meier plots and adjusted cumulative mortality plots. We identified 832 COVID-19 related deaths in the general population (online supplemental table S1). The unadjusted HR for current NSAID use was 1.26 (95% CI 1.08 to 1.47), compared with non-use in the unadjusted model (figure 2). In the multivariable-adjusted model, we observed no evidence of difference in risk (HR 0.96, 95% CI 0.80 to 1.14). There was no evidence suggesting that the HR differed by age in all adjusted models (online supplemental table S2). We did not detect deviations from the proportional hazards assumption (online supplemental table S3 and figure S6).

### Study population 2: RA/OA population

#### Patient characteristics

We included 175 495 current NSAID users and 1 533 286 non-users (table 2). A higher proportion of people aged 70+ years were included in this population than the general population. Median age was 63 years (IQR 55–71) among current users and 68 years (IQR 58–76) among non-users. Relative to current users, non-users were older at study start date. Approximately 60% of individuals were women in both groups.

Current users were more likely to be obese, more deprived, former/current smokers and to have had more primary care consultations and a prescription for proton-pump inhibitors and disease-modifying antirheumatic drugs than non-users. However, non-users were more likely to have comorbidities than current users.

### Unadjusted and multivariable results

**Figure 3:**
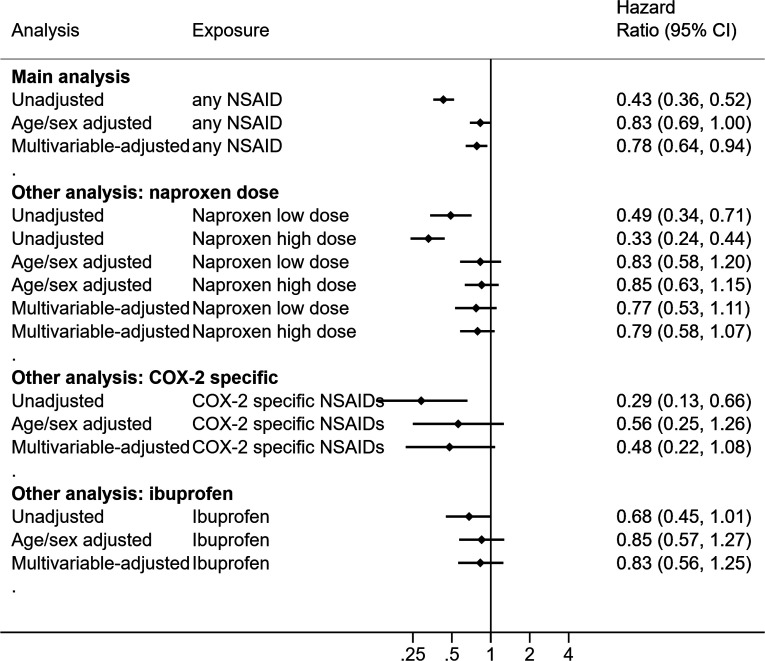
HRs of the association between current use of NSAIDs and COVID-19 related deaths in the rheumatoid arthritis or osteoarthritis population. NSAIDs, non-steroidal anti-inflammatory drugs.

Online supplemental figures S7 and S8 present time to COVID-19 related death in Kaplan-Meier plots and adjusted cumulative mortality curves, respectively. We identified 2573 COVID-19 related deaths in the RA/OA population (online supplemental table S1). The unadjusted HR for current use was 0.43 (95% CI 0.36 to 0.52), compared with non-use (figure 3). In the multivariable model, we observed a lower risk of COVID-19 related death associated with current use (HR 0.78, 95% CI 0.64 to 0.94). Post hoc analyses, after adjustment for age and sex, showed most variables had minimal impact, though adjustment for PPI moved the estimate away from the null (online supplemental table S4). There was no evidence suggesting that HR differed by age in all adjusted models. We did not detect deviations from the proportional hazards assumption (online supplemental table S3 and figure S9).

## Analyses investigating different types of NSAIDs

Online supplemental tables S5–S10 present the baseline characteristics, stratified by different types of NSAIDs. Online supplemental figures S10 and S11 present time to COVID-19 related deaths by types of NSAIDs in Kaplan-Meier plots. There was no evidence that the association with COVID-19 death varied by: (1) naproxen dose, (2) COX-specific status and (3) ibuprofen versus other NSAIDs in either study population (figures 2 and 3 and online supplemental tables S11–S13).

## Sensitivity analyses

After we excluded people who were ever prescribed aspirin, we observed no difference in risk of COVID-19 related death associated with current use compared with non-use (HR 0.84, 95% CI 0.69 to 1.02) in RA/OA population (online supplemental table S14). In the post hoc analysis when we used a directed acyclic graph (DAG) approach to select covariates, we observed a marginal decreased risk of COVID-19 in the complete case analysis, additionally adjusted for ethnicity (HR 0.79, 95% CI 0.64 to 0.99) (online supplemental table S15). The results of all other sensitivity analyses were broadly similar to those of the main analyses (online supplemental tables S16–S21).

## Quantitative bias analysis

To fully explain the multivariable-adjusted HR (0.78) or the upper bound of the 95% CI (0.94) in the RA/OA population, an unmeasured confounder would need to be associated (conditional on measured covariates) with either non-use, relative to current use or COVID-19 mortality by at least risk ratio (RR) of 1.88 (effect estimate) or 1.29 (upper bound) and with both non-use and COVID-19 mortality by at least RR of 1.28 (effect estimate) or 1.06 (upper bound) (online supplemental figure S12).

## Discussion

### Summary

Based on routinely collected data, our study showed no overall increased risk of COVID-19 related death associated with current NSAID use in adults, compared with non-use. This was consistently seen across all analyses.

In this study, we used two different populations to explore the potential impact of confounding. Current users were generally older and had more comorbidities than non-users in the general population cohort. As expected, this was associated with an increased risk of COVID-19 related death in current users compared with non-users in the unadjusted model. In contrast, current NSAID users were younger and had more comorbidities than non-users in the RA/OA population, associated with a decreased risk of COVID-19 related death in the unadjusted model. Notably, both associations were largely removed on adjustment for age. We observed a small decreased risk of COVID-19 related death among current users in the RA/OA population but not in the general population in the multivariable-adjusted models. In a post hoc analysis informed by a DAG that captures the complexity of relationships between variables, this protective effect was somewhat attenuated, suggesting it is not a robust finding and is subject to model variable selection. Moreover, our main analysis in the RA/OA population might also be subject to residual confounding. As demonstrated in quantitative bias analysis, an unmeasured confounder of only moderate strength could potentially fully explain this observed association. As we consistently found no evidence of harmful effect of NSAIDs on COVID-19 related death, using two populations provides a useful context for result interpretation.

### Findings in context

It was postulated that NSAIDs might delay diagnosis and thus clinical care by masking the symptoms of a worsening infection.^4 8–10 26^ In vivo and in vitro cellular studies show that NSAIDs weaken the immune response to pathogens by limiting the local recruitment of innate immune cells and reducing antibody synthesis, but the immunomodulatory effects of NSAIDs are not fully understood.^27 28^ Notably, these proposed mechanisms are not specific to COVID-19. Recently, it has been suggested that ibuprofen upregulates ACE 2,^29^ which has a role in binding SARS-CoV-2 to target cells and could increase the risk of developing severe COVID-19 disease through this route.^30^ Some animal studies reported that administration of soluble recombinant ACE 2 might alleviate lung injury in people with respiratory infection.^31 32^ It remains unknown whether the findings can be generalised to humans.

In line with our results, five observational studies reported no evidence of a harmful effect of NSAID use on COVID-19 severity among patients with COVID-19^33–36^ but most were of much smaller sample size and not all were general population based, limiting generalisability.^34^ A case–control study that investigated the association between renin–angiotensin–aldosterone system blockers and COVID-19 diagnosis found no association between NSAIDs and COVID-19 diagnosis.^37^ In contrast, a US cohort study reported a lower odds of mortality associated with NSAID use prior to hospitalisation among patients with COVID-19 (adjusted OR 0.56, 95% CI 0.40 to 0.82).^38^ However, patient characteristics, stratified by NSAID exposure and the covariates adjusted for, were not clear. A recent cohort study demonstrated that NSAIDs were not associated with 30-day mortality or other severe COVID-19 outcomes in Danish people who tested positive for SARS-CoV-2.^20^ This study was well conducted with robust methodology and of large sample size but it might still be subject to potential issues around selective testing for COVID-19. Furthermore, specific types of NSAIDs were not explored in the analyses, limiting the interpretation of the results.

Notably, we assessed exposure as NSAID use prior to the outbreak in England to establish who were current users, but we did not evaluate any potential therapeutic role of NSAIDs to treat patients with COVID-19. While our study mainly focused on current NSAID use for routine clinical care, there are some ongoing clinical trials investigating the role of NSAIDs in management of COVID-19. They are due to complete later this year or next year (NCT04325633^39^; NCT04382768^40^; NCT04334629^41^; and NCT04344457).^42^

### Strengths and limitations

The greatest strength of this study was the power we had to examine the association between NSAIDs and COVID-19 death, particularly on types of NSAID as our dataset included medical records from 24 million individuals. We also used two different study populations for comparisons to understand the impact of confounding by indication. The breadth of data available in primary care allows us to account for a wide range of potential confounders. We prespecified our analysis plan and have openly shared all analytical code.

We recognise possible limitations. First, we do not know whether patients truly took the medications as prescribed. Second, the supply of NSAIDs ‘over the counter’ is not captured . However, ‘over the counter’ purchases are likely to be for ibuprofen, used for acute, irregular conditions and may mean some non-users were in fact taking ibuprofen. This would tend to bias results towards the null. However, this is unlikely to impact the result in the RA/OA population as GPs in England prescribe NSAIDs for long-term conditions such as RA/OA.^43^ In our study, information on indications is not readily available; therefore, we cannot distinguish whether the NSAID use was for long-term or short-term conditions for further investigation. Notably, our results from the RA/OA population can generalise the findings to long-term NSAID users as these people receive prescriptions regularly to manage their medical condition. Additionally, we do not capture all additional medicines commonly used in the treatment of RA. In England, a small number of medicines for long-term conditions are supplied routinely by hospitals directly to patients.^44^ This includes biological treatments such as adalimumab and infliximab, and we have advocated for the release of these data but access remains restricted.^45 46^ Access to these data is important, as biological treatments might be preferentially prescribed in patients with more comorbidities, resulting in unmeasured confounding in our RA/OA population. Notably, our outcome reflected the probability of both COVID-19 infection and, once infected, COVID-19 mortality. If there was a strong harmful effect of NSAIDs on either of these endpoints, we would have observed a higher hazard of COVID-19 mortality among current users compared with non-users. However, we acknowledge that behavioural differences between our comparison groups may have led to a difference in the risk of infection, for example, if the NSAID exposed group were more risk avoidant. This could have attenuated any increased risk of harmful outcomes if differences in risk behaviour were substantial.

## Conclusions

We found no evidence of a harmful effect of routinely prescribed NSAIDs on COVID-19 related death. People currently prescribed NSAIDs for their long-term conditions should continue their treatment as part of their routine care.

### Information governance

NHS England is the data controller; TPP is the data processor; and the key researchers on OpenSAFELY are acting on behalf of NHS England. This implementation of OpenSAFELY is hosted within the TPP environment, which is accredited to the ISO 27001 information security standard and is NHS IG Toolkit compliant^47 48^; patient data have been pseudonymised for analysis and linkage using industry standard cryptographic hashing techniques; all pseudonymised datasets transmitted for linkage onto OpenSAFELY are encrypted; access to the platform is via a virtual private network connection, restricted to a small group of researchers, their specific machine and IP address; the researchers hold contracts with NHS England and only access the platform to initiate database queries and statistical models; all database activity is logged; only aggregate statistical outputs leave the platform environment following best practice for anonymisation of results such as statistical disclosure control for low cell counts.^49^ The OpenSAFELY research platform adheres to the data protection principles of the UK Data Protection Act 2018 and the EU General Data Protection Regulation 2016. In March 2020, the Secretary of State for Health and Social Care used powers under the UK Health Service (Control of Patient Information) Regulations 2002 to require organisations to process confidential patient information for the purposes of protecting public health, providing healthcare services to the public and monitoring and managing the COVID-19 outbreak and incidents of exposure.^50^ Taken together, these provide the legal bases to link patient datasets on the OpenSAFELY platform. General practices (GP), from which the primary care data are obtained, are required to share relevant health information to support the public health response to the pandemic and have been informed of the OpenSAFELY analytics platform.
