# Supplementary material for: Use of non-steroidal anti-inflammatory drugs and risk of death from COVID-19: an OpenSAFELY cohort analysis based on two cohorts
Source: Ann Rheum Dis. 2021 Jan 21:annrheumdis-2020-219517. doi: 10.1136/annrheumdis-2020-219517 (PMC7823433; doi:10.1136/annrheumdis-2020-219517)
Supplement: Supplementary file 1 [file annrheumdis-2020-219517supp001.pdf]

Supplement: Use of non-steroidal anti-inflammatory drugs and risk of death from COVID-19: an OpenSAFELY cohort analysis based on two cohorts

Contents

Figure S1. Illustration of the follow up 3
Figure S2. Directed Acyclic Graph for the association between NSAIDs and COVID-19 related deaths. 4
Figure S3. Flowchart of inclusion in both cohorts 5
Figure S4. Time to COVID-19 related death by treatment group in Kaplan-Meier plot – General population 6
Figure S5. Cumulative mortality by exposure group, standardized to the covariate distribution of the exposure group - General population 7
Table S1. Association between any oral NSAIDs and COVID-19 death 8
Table S2. Association between any oral NSAIDs and COVID-19 death, Age interaction 9
Table S3. Testing the PH assumption for COVID-19 Death in the main analysis 11
Figure S6. Schoenfeld residuals – General population 12
Figure S7. Time to COVID-19 related death by treatment group in Kaplan-Meier plot – osteoarthritis/ rheumatoid arthritis population 13
Figure S8. Cumulative mortality by exposure group, standardized to the covariate distribution of the exposure group - osteoarthritis/ rheumatoid arthritis population 14
Table S4. 1 by 1 comorbidity adjustments (after age/sex adjustments and stratified by regions) – osteoarthritis/ rheumatoid arthritis population 15
Figure S9. Schoenfeld residuals – osteoarthritis/ rheumatoid arthritis population 18
Table S5. Descriptive Characteristics in people prescribed NSAIDs in the past 3 years, stratified by naproxen dose 19
Table S6. Descriptive Characteristics in people with osteoarthritis/ rheumatoid arthritis, stratified by naproxen dose 21
Table S7. Descriptive Characteristics in people prescribed NSAIDs in the past 3 years, stratified by COX-2 specific NSAIDs 23
Table S8. Descriptive Characteristics in people with osteoarthritis/ rheumatoid arthritis, stratified by COX-2 specific NSAIDs 25
Table S9. Descriptive Characteristics in people prescribed NSAIDs in the past 3 years, stratified by ibuprofen 27
Table S10. Descriptive Characteristics in people with osteoarthritis/ rheumatoid arthritis, stratified by ibuprofen 29
Figure S10. Time to COVID-19 related death by types of NSAIDs in Kaplan-Meier plot – General population 31

Wong AYS, *et al. Ann Rheum Dis* 2021;0:1–9. doi: 10.1136/annrheumdis-2020-219517

Figure S11. Time to COVID-19 related death by types of NSAIDs in Kaplan-Meier plot – osteoarthritis/ rheumatoid arthritis population 32

Table S11. Association between low dose naproxen, high dose naproxen and other NSAIDs and COVID-19 compared with non-use of NSAIDs 33

Table S12. Association between COX-2 specific NSAIDs, non-specific NSAIDs with COVID-19 death compared with non-use of NSAIDs 34

Table S13. Association between ibuprofen, other NSAIDs with COVID-19 death compared with non-use of NSAIDs 35

Table S14. Sensitivity analysis that excluding people ever prescribed aspirin 36

Table S15. *Post-hoc* analysis that using Directed Acyclic Graph approach to select covariates 37

Table S16. Sensitivity analysis that additionally adjusting for ethnicity in the Multivariable-adjusted models 38

Table S17. Sensitivity analysis that additionally adjusted for GP consultation and A&E attendance in the Multivariable-adjusted models 39

Table S18. Sensitivity analysis that separating HbA1c no measures category from uncontrolled diabetes category in the Multivariable-adjusted models 40

Table S19. Sensitivity analysis that using 2 months to be the timeframe to identify exposure status 41

Table S20. Sensitivity analysis that removing indomethacin from exposure of interest 42

Table S21. Sensitivity analysis that not censoring people at NSAID prescription after study start date in the non-use of NSAID group as an intention-to-treat analysis 43

Figure S12. E-value for the upper 95% CI and point estimate in people with rheumatoid arthritis/ osteoarthritis 44

Figure S1. Illustration of the follow up

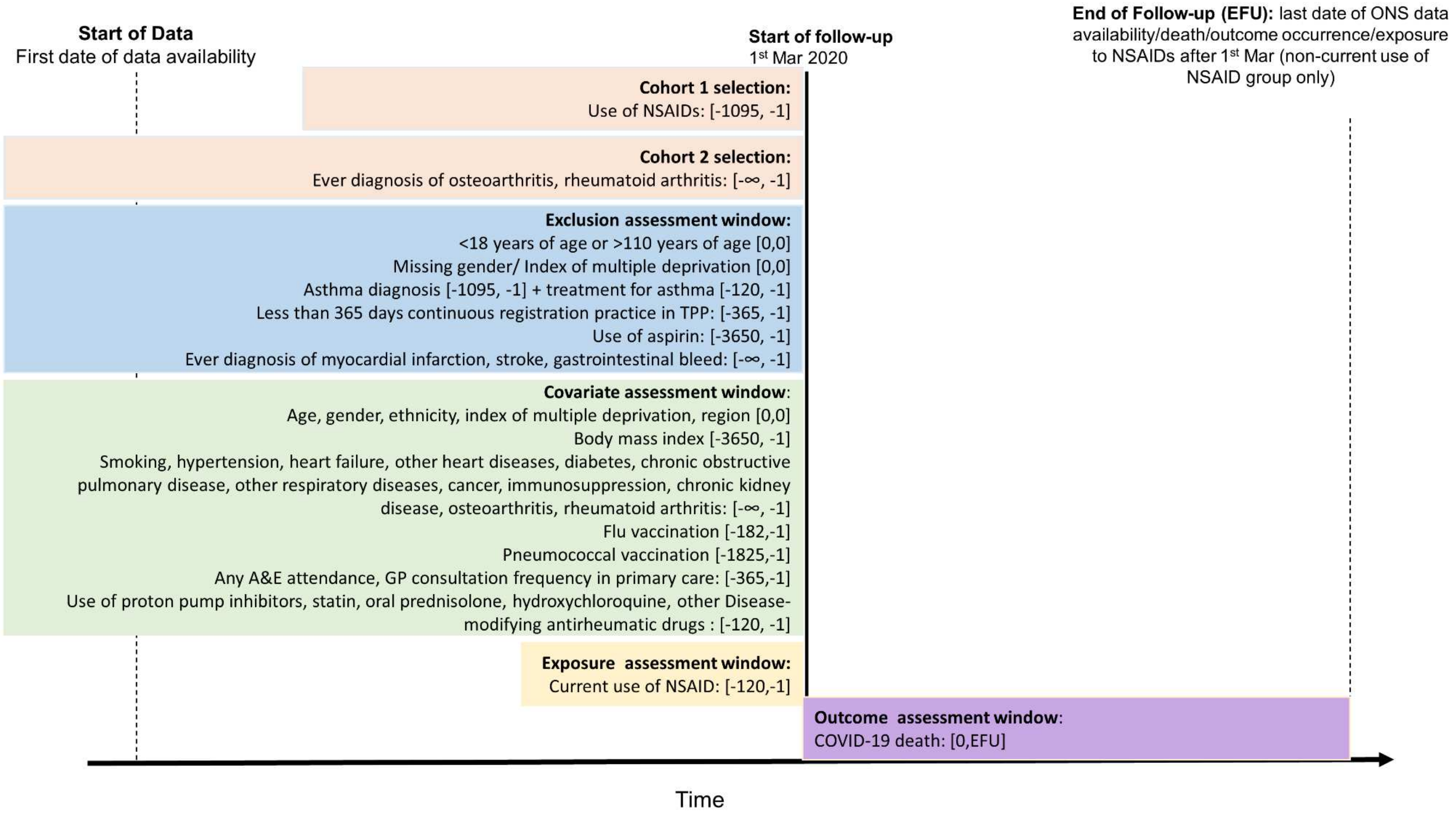

Figure S2. Directed Acyclic Graph for the association between NSAIDs and COVID-19 related deaths.

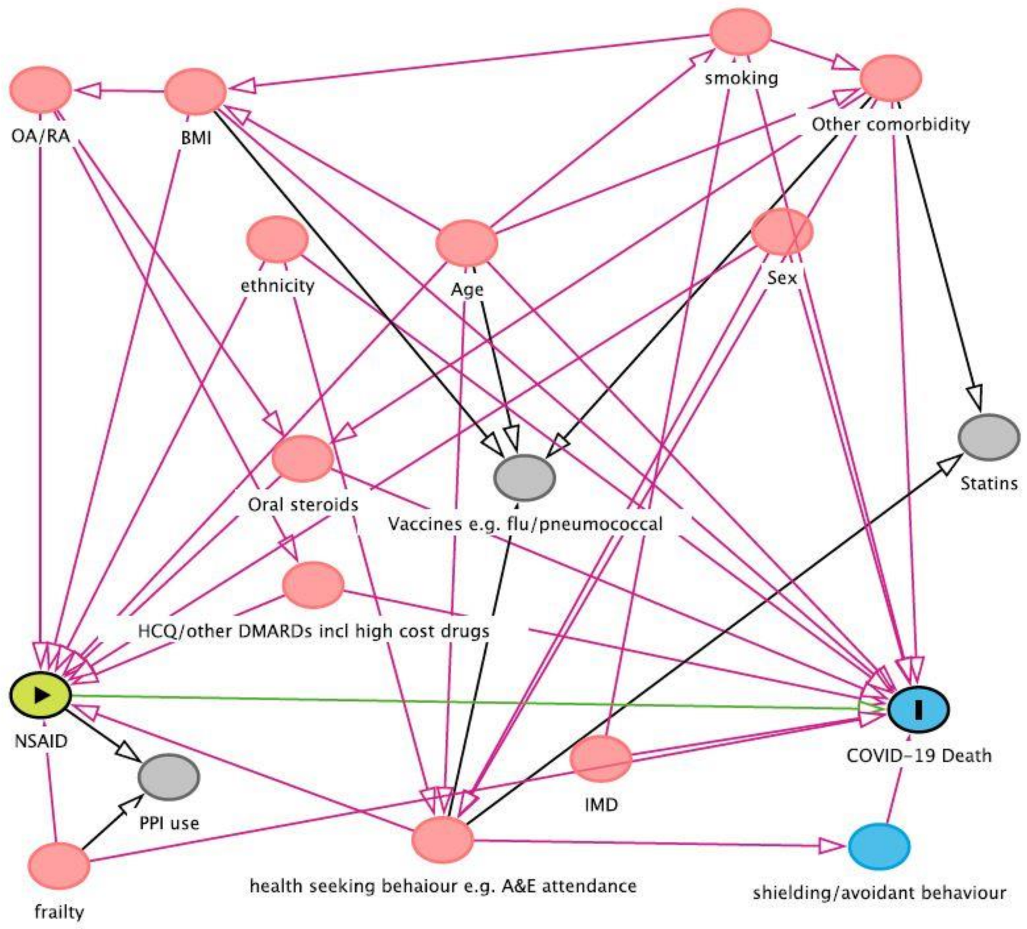

Wong AYS, *et al. Ann Rheum Dis* 2021;0:1–9. doi: 10.1136/annrheumdis-2020-219517

BMJ Publishing Group Limited (BMJ) disclaims all liability and responsibility arising from any reliance placed on this supplemental material which has been supplied by the author(s)

Figure S3. Flowchart of inclusion in both cohorts

Figure 1a. Flowchart for inclusion in people receiving NSAIDs between 1st Mar 2017 and 29th Feb 2020.

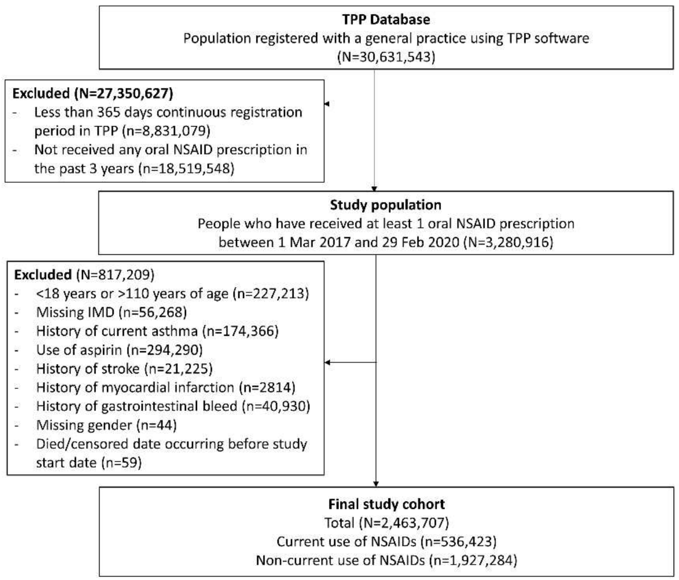

Figure 1b. Flowchart for inclusion in people with osteoarthritis or rheumatoid arthritis.

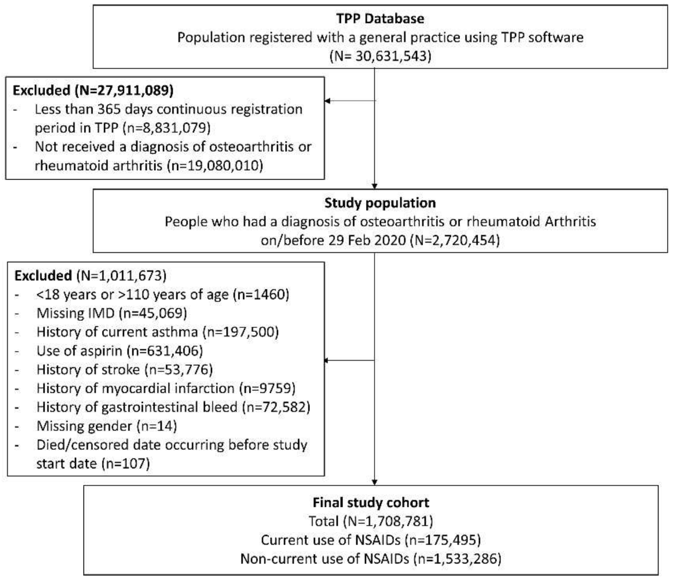

Figure S4. Time to COVID-19 related death by treatment group in Kaplan-Meier plot – General population

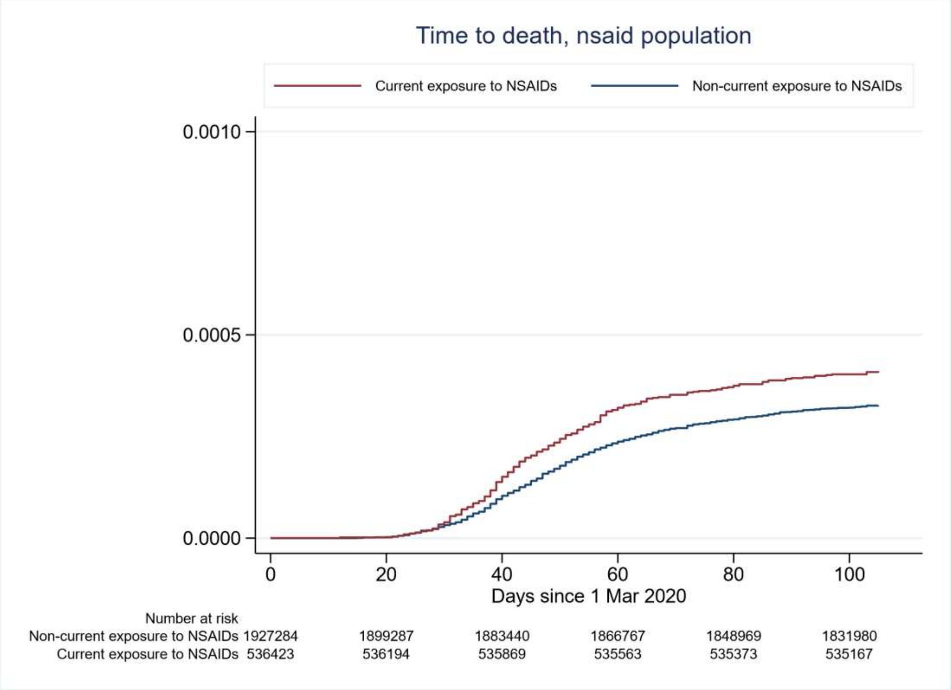

Figure S5. Cumulative mortality by exposure group, standardized to the covariate distribution of the exposure group - General population

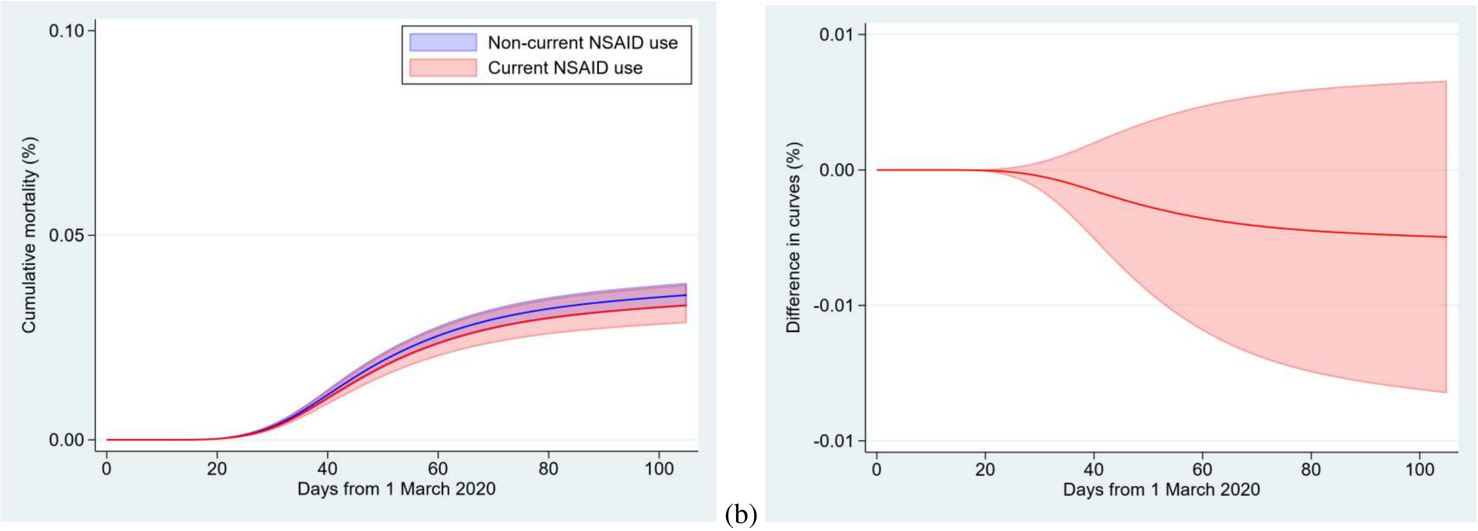

(a) (b)

Figure S5 (a) presents cumulative mortality predicted from a Royston-Parmar model including all covariates from the Multivariable-adjusted Cox model, with the baseline hazard parametrized as a 3-degrees-of-freedom cubic spline; predictions standardized to the covariate distribution of the exposure group. Figure S5 (b) presents different between two curves in Figure S4 (a).

Wong AYS, *et al. Ann Rheum Dis* 2021;0:1–9. doi: 10.1136/annrheumdis-2020-219517

Table S1. Association between any oral NSAIDs and COVID-19 death

	Number of deaths	Total person-week	Rate per 1000	Unadjusted		Age/Sex Adjusted		Multivariable-adjusted[a]	
				HR	95% CI	HR	95% CI	HR	95% CI
**Study population 1: General population (NSAIDs prescribed cohort)**									
**Non-use of NSAID**	612	28,098,800	0.02	1.00 (ref)		1.00 (ref		1.00 (ref)	
**Current use of NSAIDs**	220	8,035,882	0.03	1.26	1.08 - 1.47	1.09	0.94 - 1.28	0.96	0.80 - 1.14
**Study population 2: Rheumatoid arthritis/ osteoarthritis cohort**									
**Non-use of NSAID**	2450	22,598,389	0.11	1.00 (ref)		1.00 (ref)		1.00 (ref)	
**Current use of NSAIDs**	123	2,627,417	0.05	0.43	0.36 - 0.52	0.83	0.69 - 1.00	0.78	0.64 - 0.94

[a]Adjusted for age, sex, obesity, smoking status, deprivation, chronic kidney disease, hypertension, heart failure, other heart diseases, diabetes severity, chronic obstructive pulmonary disease, other respiratory diseases, immunosuppression, cancer, rheumatoid arthritis (study population 1), osteoarthritis (study population 1), rheumatoid arthritis/osteoarthritis/both (study population 2), statins, proton pump inhibitors, oral prednisolone, hydroxychloroquine, other DMARDS, flu vaccine and pneumococcal vaccine.

Table S2. Association between any oral NSAIDs and COVID-19 death, Age interaction

		Number of deaths	Total person-week	Rate per 1000	Unadjusted			Age/Sex Adjusted			Multivariable-adjusted[a]		
					HR	95% CI	p(inter-action)	HR	95% CI	p(inter-action)	HR	95% CI	p(inter-action)
**Study population 1: General population (NSAIDs prescribed cohort)**													
**Age group**													
**<70**							<0.01			0.10			0.29
	**Non-use of NSAID**	225	24,847,189	0.01	1.00 (ref)			1.00 (ref)			1.00 (ref)		
	**Current use of NSAIDs**	98	6,867,811	0.01	1.58	1.24 - 2.00		1.28	1.01 - 1.63		1.06	0.82 - 1.36	
**≥70**													
	**Non-use of NSAID**	387	3,251,613	0.12	1.00 (ref)			1.00 (ref)			1.00 (ref)		
	**Current use of NSAIDs**	122	1,168,071	0.1	0.88	0.72 - 1.08		0.98	0.80 - 1.21		0.89	0.72 - 1.11	
**Study population 2: Rheumatoid arthritis/ osteoarthritis cohort**													
**Age group**													
**<70**							<0.01			0.03			0.16
	**Non-use of NSAID**	256	12,357,511	0.02	1.00 (ref)			1.00 (ref)			1.00 (ref)		
	**Current use of NSAIDs**	41	1,837,108	0.02	1.08	0.77 - 1.50		1.15	0.82 - 1.59		0.95	0.68 - 1.33	
**≥70**													
	**Non-use of NSAID**	2194	10,240,877	0.21	1.00 (ref)			1.00 (ref)			1.00 (ref)		
	**Current use of NSAIDs**	82	790,308	0.10	0.48	0.39 - 0.60		0.74	0.59 - 0.92		0.71	0.57 - 0.90	

[a]Adjusted for age, sex, obesity, smoking status, deprivation, chronic kidney disease, hypertension, heart failure, other heart diseases, diabetes severity, chronic obstructive pulmonary disease, other respiratory diseases, immunosuppression, cancer, rheumatoid arthritis (study population 1), osteoarthritis (study

population 1), rheumatoid arthritis/osteoarthritis/both (study population 2), statins, proton pump inhibitors, oral prednisolone, hydroxychloroquine, other DMARDS, flu vaccine and pneumococcal vaccine.

Table S3. Testing the PH assumption for COVID-19 Death in the main analysis

	Unadjusted	Age/Sex Adjusted	Multivariable-adjusted[a]
	p-value	p-value	p-value
**Study population 1: General population (NSAIDs prescribed cohort)**			
Treatment Exposure	0.18	0.25	0.20
**Study population 2: Rheumatoid arthritis/ osteoarthritis cohort**			
Treatment Exposure	0.15	0.72	0.87

[a]Adjusted for age, sex, obesity, smoking status, deprivation, chronic kidney disease, hypertension, heart failure, other heart diseases, diabetes severity, chronic obstructive pulmonary disease, other respiratory diseases, immunosuppression, cancer, rheumatoid arthritis (study population 1), osteoarthritis (study population 1), rheumatoid arthritis/osteoarthritis/both (study population 2), statins, proton pump inhibitors, oral prednisolone, hydroxychloroquine, other DMARDS, flu vaccine and pneumococcal vaccine.

Wong AYS, *et al. Ann Rheum Dis* 2021;0:1–9. doi: 10.1136/annrheumdis-2020-219517

Figure S6. Schoenfeld residuals – General population

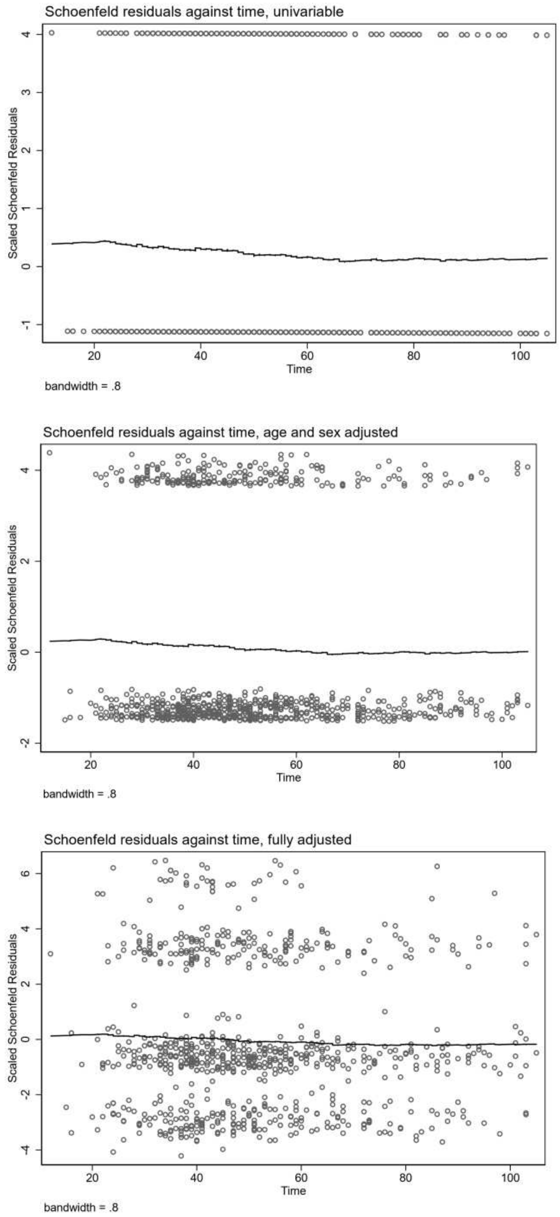

Wong AYS, *et al. Ann Rheum Dis* 2021;0:1–9. doi: 10.1136/annrheumdis-2020-219517

Figure S7. Time to COVID-19 related death by treatment group in Kaplan-Meier plot – osteoarthritis/ rheumatoid arthritis population

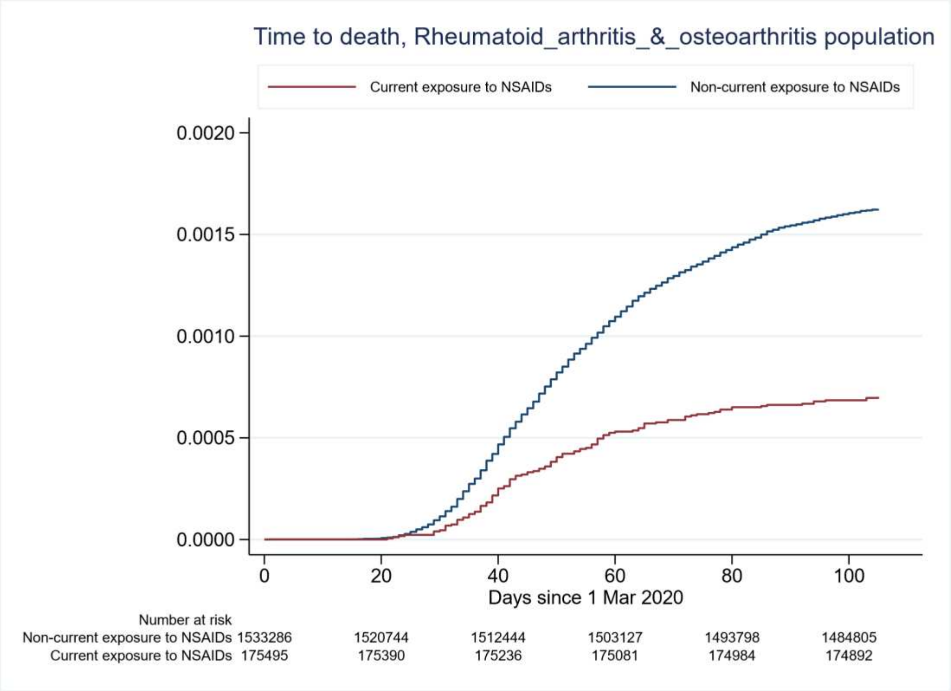

Wong AYS, *et al. Ann Rheum Dis* 2021;0:1–9. doi: 10.1136/annrheumdis-2020-219517

Figure S8. Cumulative mortality by exposure group, standardized to the covariate distribution of the exposure group - osteoarthritis/ rheumatoid arthritis population

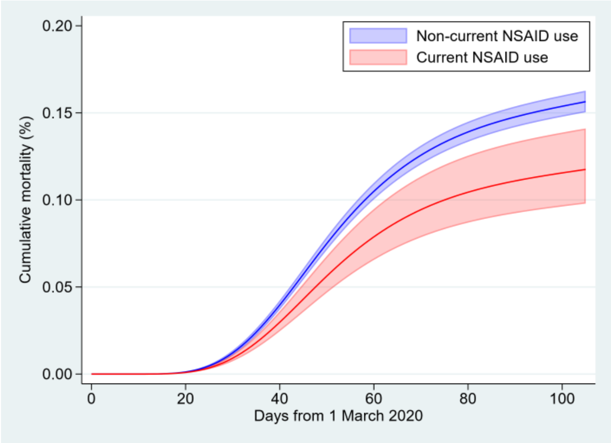

(a)

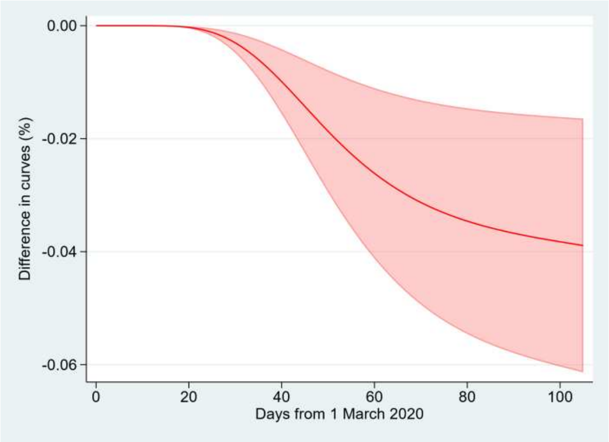

(b)

Figure S8 (a) presents cumulative mortality predicted from a Royston-Parmar model including all covariates from the Multivariable-adjusted Cox model, with the baseline hazard parametrized as a 2-degrees-of-freedom cubic spline; predictions standardized to the covariate distribution of the exposure group. Figure S8 (b) presents different between two curves in Figure S7 (a).

Wong AYS, *et al. Ann Rheum Dis* 2021;0:1–9. doi: 10.1136/annrheumdis-2020-219517

Table S4. 1 by 1 comorbidity adjustments (after age/sex adjustments and stratified by regions) – osteoarthritis/ rheumatoid arthritis population

	HR	95% CI
Smoking status		
non-current NSAID use	1.00 (ref)	
current NSAID use	0.86	0.72 - 1.03
Index of Multiple Deprivation (IMD)		
non-current NSAID use	1.00 (ref)	
current NSAID use	0.87	0.72 - 1.04
Chronic kidney disease		
non-current NSAID use	1.00 (ref)	
current NSAID use	0.87	0.73 - 1.05
Diagnosed hypertension		
non-current NSAID use	1.00 (ref)	
current NSAID use	0.87	0.72 - 1.04
Heart Failure		
non-current NSAID use	1.00 (ref)	
current NSAID use	0.88	0.74 - 1.06
Other Heart Diseases		
non-current NSAID use	1.00 (ref)	
current NSAID use	0.87	0.73 - 1.05
COPD		
non-current NSAID use	1.00 (ref)	
current NSAID use	0.87	0.73 - 1.05
Other Respiratory Diseases		
non-current NSAID use	1.00 (ref)	
current NSAID use	0.87	0.73 - 1.05
Immunosuppressed		
non-current NSAID use	1.00 (ref)	
current NSAID use	0.87	0.72 - 1.04
Cancer		

non-current NSAID use	1.00 (ref)	
current NSAID use	0.87	0.72 - 1.04
Rheumatoid arthritis/osteoarthritis/both		
non-current NSAID use	1.00 (ref)	
current NSAID use	0.84	0.70 - 1.01
Recent Statin		
non-current NSAID use	1.00 (ref)	
current NSAID use	0.87	0.72 - 1.04
Recent PPIs		
non-current NSAID use	1.00 (ref)	
current NSAID use	0.71	0.59 - 0.86
Recent oral prednisolone		
non-current NSAID use	1.00 (ref)	
current NSAID use	0.86	0.72 - 1.03
Recent hydroxychloroquine		
non-current NSAID use	1.00 (ref)	
current NSAID use	0.86	0.71 - 1.03
Recent Other DMARDs use		
non-current NSAID use	1.00 (ref)	
current NSAID use	0.85	0.71 - 1.02
Flu vaccine		
non-current NSAID use	1.00 (ref)	
current NSAID use	0.86	0.72 - 1.04
Pneumococcal Vaccine		
non-current NSAID use	1.00 (ref)	
current NSAID use	0.86	0.72 - 1.04
Diabetes Severity		
non-current NSAID use	1.00 (ref)	
current NSAID use	0.88	0.73 - 1.06
GP consultation in last year (binary)		
non-current NSAID use	1.00 (ref)	

Wong AYS, *et al. Ann Rheum Dis* 2021;0:1–9. doi: 10.1136/annrheumdis-2020-219517

current NSAID use	0.88	0.73 - 1.05
A&E attendance rate in last year (binary)		
non-current NSAID use	1.00 (ref)	
current NSAID use	0.86	0.71 - 1.03

Figure S9. Schoenfeld residuals – osteoarthritis/ rheumatoid arthritis population

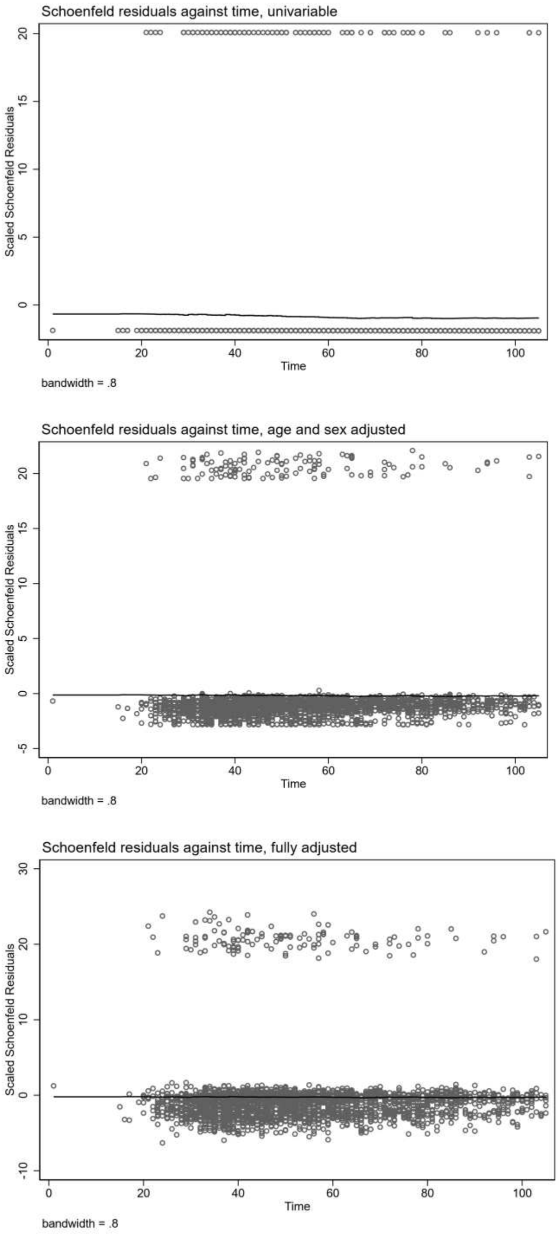

Wong AYS, *et al. Ann Rheum Dis* 2021;0:1–9. doi: 10.1136/annrheumdis-2020-219517

Table S5. Descriptive Characteristics in people prescribed NSAIDs in the past 3 years, stratified by naproxen dose

	Non-use of NSAIDs	Current use of naproxen low dose	Current use of naproxen high dose	Current use of other NSAIDs
**Total**	1,927,284	109,656	292,365	134,402
**Age as of 1st March 2020**				
18-<40	598,513 (31.1)	23,522 (21.5)	69,802 (23.9)	22,534 (16.8)
40-<50	397,201 (20.6)	19,265 (17.6)	62,733 (21.5)	21,078 (15.7)
50-<60	423,937 (22.0)	25,271 (23.0)	78,164 (26.7)	29,631 (22.0)
60-<70	283,639 (14.7)	22,527 (20.5)	53,639 (18.3)	30,039 (22.4)
70-<80	169,281 (8.8)	14,762 (13.5)	23,904 (8.2)	23,555 (17.5)
80+	54,713 (2.8)	4,309 (3.9)	4,123 (1.4)	7,565 (5.6)
**Sex**				
Female	1,093,581 (56.7)	68,877 (62.8)	162,181 (55.5)	86,283 (64.2)
**Body mass index**				
<18.5	26,435 (1.4)	1,398 (1.3)	2,756 (0.9)	1,887 (1.4)
18.5-24.9	484,862 (25.2)	24,845 (22.7)	60,379 (20.7)	29,433 (21.9)
25-29.9	577,087 (29.9)	33,230 (30.3)	86,684 (29.6)	39,659 (29.5)
30-34.9	333,254 (17.3)	21,537 (19.6)	57,949 (19.8)	26,828 (20.0)
35-39.9	138,059 (7.2)	9,848 (9.0)	27,377 (9.4)	13,181 (9.8)
40+	71,503 (3.7)	5,773 (5.3)	16,771 (5.7)	7,894 (5.9)
Missing	296,084 (15.4)	13,025 (11.9)	40,449 (13.8)	15,520 (11.5)
**Ethnicity**				
White	1,236,854 (64.2)	72,279 (65.9)	195,500 (66.9)	89,872 (66.9)
Mixed	20,556 (1.1)	1,005 (0.9)	2,726 (0.9)	965 (0.7)
Asian or Asian British	151,533 (7.9)	8,711 (7.9)	17,431 (6.0)	6,868 (5.1)
Black	49,618 (2.6)	2,244 (2.0)	6,326 (2.2)	1,957 (1.5)
Other	30,214 (1.6)	1,517 (1.4)	3,969 (1.4)	1,439 (1.1)
Missing	438,509 (22.8)	23,900 (21.8)	66,413 (22.7)	33,301 (24.8)
**Index of Multiple Deprivation**				
1 (least deprived)	388,369 (20.2)	21,688 (19.8)	57,704 (19.7)	28,149 (20.9)
2	387,428 (20.1)	21,656 (19.7)	59,074 (20.2)	28,267 (21.0)
3	382,357 (19.8)	22,087 (20.1)	57,616 (19.7)	27,923 (20.8)
4	384,598 (20.0)	22,235 (20.3)	58,381 (20.0)	25,982 (19.3)
5 (most deprived)	384,532 (20.0)	21,990 (20.1)	59,590 (20.4)	24,081 (17.9)
**Smoking status**				
Never	841,256 (43.6)	46,750 (42.6)	117,900 (40.3)	55,643 (41.4)
Former	665,068 (34.5)	41,757 (38.1)	110,983 (38.0)	54,614 (40.6)
Current	389,340 (20.2)	19,946 (18.2)	60,430 (20.7)	22,882 (17.0)
Missing	31,620 (1.6)	1,203 (1.1)	3,052 (1.0)	1,263 (0.9)
**Comorbidities**				
Hypertension	353,803 (18.4)	27,674 (25.2)	60,595 (20.7)	39,809 (29.6)
Heart Failure	9,512 (0.5)	538 (0.5)	1,008 (0.3)	887 (0.7)
Other Heart Disease	27,881 (1.4)	1,876 (1.7)	4,166 (1.4)	2,684 (2.0)
Diabetes				
Controlled (HbA1c <	122,653 (6.4)	9,464 (8.6)	20,436 (7.0)	12,232 (9.1)

Wong AYS, *et al. Ann Rheum Dis* 2021;0:1–9. doi: 10.1136/annrheumdis-2020-219517

58mmols/mol)				
Uncontrolled (HbA1c ≥ 58mmols/mol)	50,268 (2.6)	3,424 (3.1)	8,320 (2.8)	4,760 (3.5)
HbA1c not measured	4,536 (0.2)	257 (0.2)	680 (0.2)	366 (0.3)
COPD	42,636 (2.2)	3,280 (3.0)	7,046 (2.4)	5,109 (3.8)
Other respiratory diseases	17,270 (0.9)	1,272 (1.2)	2,610 (0.9)	2,312 (1.7)
Cancer	95,315 (4.9)	6,759 (6.2)	14,261 (4.9)	11,108 (8.3)
Immunosuppression	9,285 (0.5)	566 (0.5)	1,392 (0.5)	960 (0.7)
Chronic kidney disease	51,642 (2.7)	4,057 (3.7)	6,382 (2.2)	7,131 (5.3)
Osteoarthritis	367,954 (19.1)	34,012 (31.0)	77,224 (26.4)	51,440 (38.3)
Rheumatoid arthritis	28,690 (1.5)	3,589 (3.3)	8,158 (2.8)	9,779 (7.3)
**GP consultations**				
Median, IQR	5 (2-10)	8 (4-13)	7 (4-12)	8 (4-14)
Min, Max	0, 626	0, 400	0, 560	0, 576
**A&E attendance**				
Median, IQR	0 (0-0)	0 (0-0)	0 (0-0)	0 (0-0)
Min, Max	0, 118	0, 56	0, 152	0, 67
**Vaccination**				
Flu	435,383 (22.6)	35,307 (32.2)	71,987 (24.6)	54,788 (40.8)
Pneumococcal	116,464 (6.0)	9,667 (8.8)	20,502 (7.0)	14,733 (11.0)
**Medications**				
Statin	223,221 (11.6)	19,455 (17.7)	40,088 (13.7)	27,626 (20.6)
Proton pump inhibitors	268,934 (14.0)	72,192 (65.8)	188,428 (64.4)	81,646 (60.7)
Oral prednisolone	39,081 (2.0)	3,128 (2.9)	7,291 (2.5)	5,665 (4.2)
Hydroxychloroquine	8,074 (0.4)	1,132 (1.0)	2,580 (0.9)	2,968 (2.2)
Other DMARDs	20,770 (1.1)	2,739 (2.5)	6,159 (2.1)	7,959 (5.9)

Abbreviations: COPD, Chronic obstructive pulmonary disease; DMARDs, Disease-modifying antirheumatic drugs.

Table S6. Descriptive Characteristics in people with osteoarthritis/ rheumatoid arthritis, stratified by naproxen dose

	Non-use of NSAIDs	Current use of naproxen low dose	Current use of naproxen high dose	Current use of other NSAIDs
**Total**	1,533,286	36,132	82,305	57,058
**Age as of 1st March 2020**				
18-<40	32,958 (2.1)	752 (2.1)	2,394 (2.9)	1,287 (2.3)
40-<50	97,870 (6.4)	2,705 (7.5)	8,929 (10.8)	4,179 (7.3)
50-<60	292,186 (19.1)	8,123 (22.5)	25,449 (30.9)	11,825 (20.7)
60-<70	416,489 (27.2)	11,904 (32.9)	27,384 (33.3)	17,659 (30.9)
70-<80	436,477 (28.5)	9,592 (26.5)	15,312 (18.6)	16,446 (28.8)
80+	257,306 (16.8)	3,056 (8.5)	2,837 (3.4)	5,662 (9.9)
**Sex**				
Female	951,417 (62.1)	23,929 (66.2)	48,559 (59.0)	38,038 (66.7)
**Body mass index**				
<18.5	19,616 (1.3)	278 (0.8)	429 (0.5)	553 (1.0)
18.5-24.9	379,233 (24.7)	6,925 (19.2)	13,463 (16.4)	11,143 (19.5)
25-29.9	518,602 (33.8)	11,739 (32.5)	25,791 (31.3)	17,857 (31.3)
30-34.9	298,505 (19.5)	8,307 (23.0)	19,540 (23.7)	12,666 (22.2)
35-39.9	119,286 (7.8)	3,919 (10.8)	9,815 (11.9)	6,328 (11.1)
40+	58,801 (3.8)	2,296 (6.4)	6,273 (7.6)	3,827 (6.7)
Missing	139,243 (9.1)	2,668 (7.4)	6,994 (8.5)	4,684 (8.2)
**Ethnicity**				
White	1,095,982 (71.5)	25,805 (71.4)	58,884 (71.5)	40,384 (70.8)
Mixed	6,563 (0.4)	186 (0.5)	430 (0.5)	214 (0.4)
Asian or Asian British	51,587 (3.4)	1,862 (5.2)	3,096 (3.8)	2,011 (3.5)
Black	17,645 (1.2)	431 (1.2)	1,215 (1.5)	460 (0.8)
Other	10,916 (0.7)	274 (0.8)	603 (0.7)	364 (0.6)
Missing	350,593 (22.9)	7,574 (21.0)	18,077 (22.0)	13,625 (23.9)
**Index of Multiple Deprivation**				
1 (least deprived)	313,701 (20.5)	6,365 (17.6)	13,976 (17.0)	10,456 (18.3)
2	309,372 (20.2)	6,753 (18.7)	15,239 (18.5)	10,954 (19.2)
3	307,669 (20.1)	7,067 (19.6)	15,869 (19.3)	11,661 (20.4)
4	303,859 (19.8)	7,607 (21.1)	17,210 (20.9)	11,865 (20.8)
5 (most deprived)	298,685 (19.5)	8,340 (23.1)	20,011 (24.3)	12,122 (21.2)
**Smoking status**				
Never	672,833 (43.9)	14,961 (41.4)	32,448 (39.4)	22,874 (40.1)
Former	692,164 (45.1)	16,693 (46.2)	37,422 (45.5)	26,868 (47.1)
Current	164,464 (10.7)	4,423 (12.2)	12,263 (14.9)	7,227 (12.7)
Missing	3,825 (0.2)	55 (0.2)	172 (0.2)	89 (0.2)
**Comorbidities**				
Hypertension	625,247 (40.8)	14,412 (39.9)	28,162 (34.2)	23,524 (41.2)
Heart Failure	36,888 (2.4)	317 (0.9)	509 (0.6)	587 (1.0)
Other Heart Disease	57,976 (3.8)	888 (2.5)	1,810 (2.2)	1,504 (2.6)
Diabetes				
Controlled (HbA1c <	177,397 (11.6)	4,401 (12.2)	8,574 (10.4)	6,560 (11.5)

Wong AYS, *et al. Ann Rheum Dis* 2021;0:1–9. doi: 10.1136/annrheumdis-2020-219517

58mmols/mol)				
Uncontrolled (HbA1c ≥ 58mmols/mol)	58,452 (3.8)	1,284 (3.6)	2,878 (3.5)	2,124 (3.7)
HbA1c not measured	3,695 (0.2)	92 (0.3)	177 (0.2)	150 (0.3)
COPD	85,858 (5.6)	1,826 (5.1)	3,453 (4.2)	3,094 (5.4)
Other respiratory diseases	38,248 (2.5)	729 (2.0)	1,281 (1.6)	1,425 (2.5)
Cancer	174,647 (11.4)	3,395 (9.4)	6,403 (7.8)	6,142 (10.8)
Immunosuppression	8,498 (0.6)	185 (0.5)	415 (0.5)	409 (0.7)
Chronic kidney disease	164,985 (10.8)	2,547 (7.0)	3,700 (4.5)	4,901 (8.6)
Osteoarthritis	1,473,833 (96.1)	34,012 (94.1)	77,224 (93.8)	51,440 (90.2)
Rheumatoid arthritis	95,056 (6.2)	3,589 (9.9)	8,158 (9.9)	9,779 (17.1)
**GP consultations**				
Median, IQR	6 (3-11)	8 (5-14)	8 (5-14)	8 (5-14)
Min, Max	0, 468	0, 360	0, 318	0, 260
**A&E attendance**				
Median, IQR	0 (0-0)	0 (0-0)	0 (0-0)	0 (0-0)
Min, Max	0, 71	0, 26	0, 36	0, 63
**Vaccination**				
Flu	806,064 (52.6)	18,929 (52.4)	35,280 (42.9)	32,306 (56.6)
Pneumococcal	193,145 (12.6)	5,327 (14.7)	10,665 (13.0)	8,752 (15.3)
**Medications**				
Statin	415,120 (27.1)	10,504 (29.1)	19,976 (24.3)	16,540 (29.0)
Proton pump inhibitors	371,464 (24.2)	29,365 (81.3)	65,940 (80.1)	41,875 (73.4)
Oral prednisolone	61,256 (4.0)	1,519 (4.2)	3,312 (4.0)	3,434 (6.0)
Hydroxychloroquine	16,783 (1.1)	851 (2.4)	1,958 (2.4)	2,295 (4.0)
Other DMARDs	48,819 (3.2)	2,098 (5.8)	4,616 (5.6)	6,039 (10.6)

Abbreviations: COPD,7 Chronic obstructive pulmonary disease; DMARDs, Disease-modifying antirheumatic drugs

Wong AYS, *et al. Ann Rheum Dis* 2021;0:1–9. doi: 10.1136/annrheumdis-2020-219517

Table S7. Descriptive Characteristics in people prescribed NSAIDs in the past 3 years, stratified by COX-2 specific NSAIDs

	Non-use of NSAIDs	Current use of COX-2 specific	Current use of non-specific NSAIDs
**Total**	1,927,284	24,808	511,615
**Age as of 1st March 2020**			
18-<40	598,513 (31.1)	3,210 (12.9)	112,648 (22.0)
40-<50	397,201 (20.6)	4,255 (17.2)	98,821 (19.3)
50-<60	423,937 (22.0)	6,612 (26.7)	126,454 (24.7)
60-<70	283,639 (14.7)	5,748 (23.2)	100,457 (19.6)
70-<80	169,281 (8.8)	3,841 (15.5)	58,380 (11.4)
80+	54,713 (2.8)	1,142 (4.6)	14,855 (2.9)
**Sex**			
Female	1,093,581 (56.7)	15,475 (62.4)	301,866 (59.0)
**Body mass index**			
<18.5	26,435 (1.4)	274 (1.1)	5,767 (1.1)
18.5-24.9	484,862 (25.2)	5,350 (21.6)	109,307 (21.4)
25-29.9	577,087 (29.9)	7,615 (30.7)	151,958 (29.7)
30-34.9	333,254 (17.3)	5,045 (20.3)	101,269 (19.8)
35-39.9	138,059 (7.2)	2,536 (10.2)	47,870 (9.4)
40+	71,503 (3.7)	1,438 (5.8)	29,000 (5.7)
Missing	296,084 (15.4)	2,550 (10.3)	66,444 (13.0)
**Ethnicity**			
White	1,236,854 (64.2)	17,283 (69.7)	340,368 (66.5)
Mixed	20,556 (1.1)	130 (0.5)	4,566 (0.9)
Asian or Asian British	151,533 (7.9)	988 (4.0)	32,022 (6.3)
Black	49,618 (2.6)	208 (0.8)	10,319 (2.0)
Other	30,214 (1.6)	230 (0.9)	6,695 (1.3)
Missing	438,509 (22.8)	5,969 (24.1)	117,645 (23.0)
**Index of Multiple Deprivation**			
1 (least deprived)	388,369 (20.2)	5,536 (22.3)	102,005 (19.9)
2	387,428 (20.1)	5,636 (22.7)	103,361 (20.2)
3	382,357 (19.8)	5,221 (21.0)	102,405 (20.0)
4	384,598 (20.0)	4,528 (18.3)	102,070 (20.0)
5 (most deprived)	384,532 (20.0)	3,887 (15.7)	101,774 (19.9)
**Smoking status**			
Never	841,256 (43.6)	9,803 (39.5)	210,490 (41.1)
Former	665,068 (34.5)	10,680 (43.1)	196,674 (38.4)
Current	389,340 (20.2)	4,225 (17.0)	99,033 (19.4)
Missing	31,620 (1.6)	100 (0.4)	5,418 (1.1)
**Comorbidities**			
Hypertension	353,803 (18.4)	7,530 (30.4)	120,548 (23.6)
Heart Failure	9,512 (0.5)	174 (0.7)	2,259 (0.4)
Other Heart Disease	27,881 (1.4)	502 (2.0)	8,224 (1.6)
Diabetes			

Wong AYS, *et al. Ann Rheum Dis* 2021;0:1–9. doi: 10.1136/annrheumdis-2020-219517

Controlled (HbA1c < 58mmols/mol)	122,653 (6.4)	2,185 (8.8)	39,947 (7.8)
Uncontrolled (HbA1c ≥ 58mmols/mol)	50,268 (2.6)	779 (3.1)	15,725 (3.1)
HbA1c not measured	4,536 (0.2)	72 (0.3)	1,231 (0.2)
COPD	42,636 (2.2)	983 (4.0)	14,452 (2.8)
Other respiratory diseases	17,270 (0.9)	461 (1.9)	5,733 (1.1)
Cancer	95,315 (4.9)	1,837 (7.4)	30,291 (5.9)
Immunosuppression	9,285 (0.5)	186 (0.7)	2,732 (0.5)
Chronic kidney disease	51,642 (2.7)	1,477 (6.0)	16,093 (3.1)
Osteoarthritis	367,954 (19.1)	10,737 (43.3)	151,939 (29.7)
Rheumatoid arthritis	28,690 (1.5)	3,205 (12.9)	18,321 (3.6)
**GP consultations**			
Median, IQR	5 (2-10)	9 (5-15)	7 (4-13)
Min, Max	0, 626	0, 565	0, 576
**A&E attendance**			
Median, IQR	0 (0-0)	0 (0-0)	0 (0-0)
Min, Max	0, 118	0, 49	0, 152
**Vaccination**			
Flu	435,383 (22.6)	10,513 (42.4)	151,569 (29.6)
Pneumococcal	116,464 (6.0)	3,312 (13.4)	41,590 (8.1)
**Medications**			
Statin	223,221 (11.6)	4,934 (19.9)	82,235 (16.1)
Proton pump inhibitors	268,934 (14.0)	16,416 (66.2)	325,850 (63.7)
Oral prednisolone	39,081 (2.0)	1,494 (6.0)	14,590 (2.9)
Hydroxychloroquine	8,074 (0.4)	1,040 (4.2)	5,640 (1.1)
Other DMARDs	20,770 (1.1)	2,856 (11.5)	14,001 (2.7)

Abbreviations: COPD, Chronic obstructive pulmonary disease; DMARDs, Disease-modifying antirheumatic drugs.

Table S8. Descriptive Characteristics in people with osteoarthritis/ rheumatoid arthritis, stratified by COX-2 specific NSAIDs

	Non-use of NSAIDs	Current use of COX-2 specific	Current use of non-specific NSAIDs
**Total**	1,533,286	12,559	162,936
**Age as of 1st March 2020**			
18-<40	32,958 (2.1)	459 (3.7)	3,974 (2.4)
40-<50	97,870 (6.4)	1,310 (10.4)	14,503 (8.9)
50-<60	292,186 (19.1)	3,158 (25.1)	42,239 (25.9)
60-<70	416,489 (27.2)	3,818 (30.4)	53,129 (32.6)
70-<80	436,477 (28.5)	2,894 (23.0)	38,456 (23.6)
80+	257,306 (16.8)	920 (7.3)	10,635 (6.5)
**Sex**			
Female	951,417 (62.1)	8,725 (69.5)	101,801 (62.5)
**Body mass index**			
<18.5	19,616 (1.3)	118 (0.9)	1,142 (0.7)
18.5-24.9	379,233 (24.7)	2,530 (20.1)	29,001 (17.8)
25-29.9	518,602 (33.8)	3,938 (31.4)	51,449 (31.6)
30-34.9	298,505 (19.5)	2,759 (22.0)	37,754 (23.2)
35-39.9	119,286 (7.8)	1,427 (11.4)	18,635 (11.4)
40+	58,801 (3.8)	853 (6.8)	11,543 (7.1)
Missing	139,243 (9.1)	934 (7.4)	13,412 (8.2)
**Ethnicity**			
White	1,095,982 (71.5)	8,939 (71.2)	116,134 (71.3)
Mixed	6,563 (0.4)	37 (0.3)	793 (0.5)
Asian or Asian British	51,587 (3.4)	434 (3.5)	6,535 (4.0)
Black	17,645 (1.2)	99 (0.8)	2,007 (1.2)
Other	10,916 (0.7)	99 (0.8)	1,142 (0.7)
Missing	350,593 (22.9)	2,951 (23.5)	36,325 (22.3)
**Index of Multiple Deprivation**			
1 (least deprived)	313,701 (20.5)	2,309 (18.4)	28,488 (17.5)
2	309,372 (20.2)	2,406 (19.2)	30,540 (18.7)
3	307,669 (20.1)	2,606 (20.8)	31,991 (19.6)
4	303,859 (19.8)	2,567 (20.4)	34,115 (20.9)
5 (most deprived)	298,685 (19.5)	2,671 (21.3)	37,802 (23.2)
**Smoking status**			
Never	672,833 (43.9)	4,957 (39.5)	65,326 (40.1)
Former	692,164 (45.1)	5,887 (46.9)	75,096 (46.1)
Current	164,464 (10.7)	1,695 (13.5)	22,218 (13.6)
Missing	3,825 (0.2)	20 (0.2)	296 (0.2)
**Comorbidities**			
Hypertension	625,247 (40.8)	4,803 (38.2)	61,295 (37.6)
Heart Failure	36,888 (2.4)	123 (1.0)	1,290 (0.8)
Other Heart Disease	57,976 (3.8)	328 (2.6)	3,874 (2.4)
Diabetes			

Wong AYS, *et al. Ann Rheum Dis* 2021;0:1–9. doi: 10.1136/annrheumdis-2020-219517

Controlled (HbA1c < 58mmols/mol)	177,397 (11.6)	1,347 (10.7)	18,188 (11.2)
Uncontrolled (HbA1c ≥ 58mmols/mol)	58,452 (3.8)	411 (3.3)	5,875 (3.6)
HbA1c not measured	3,695 (0.2)	38 (0.3)	381 (0.2)
COPD	85,858 (5.6)	666 (5.3)	7,707 (4.7)
Other respiratory diseases	38,248 (2.5)	337 (2.7)	3,098 (1.9)
Cancer	174,647 (11.4)	1,151 (9.2)	14,789 (9.1)
Immunosuppression	8,498 (0.6)	104 (0.8)	905 (0.6)
Chronic kidney disease	164,985 (10.8)	1,095 (8.7)	10,053 (6.2)
Osteoarthritis	1,473,833 (96.1)	10,737 (85.5)	151,939 (93.3)
Rheumatoid arthritis	95,056 (6.2)	3,205 (25.5)	18,321 (11.2)
**GP consultations**			
Median, IQR	6 (3-11)	9 (5-16)	8 (5-14)
Min, Max	0, 468	0, 225	0, 360
**A&E attendance**			
Median, IQR	0 (0-0)	0 (0-0)	0 (0-0)
Min, Max	0, 71	0, 24	0, 63
**Vaccination**			
Flu	806,064 (52.6)	6,897 (54.9)	79,618 (48.9)
Pneumococcal	193,145 (12.6)	2,068 (16.5)	22,676 (13.9)
**Medications**			
Statin	415,120 (27.1)	3,240 (25.8)	43,780 (26.9)
Proton pump inhibitors	371,464 (24.2)	9,038 (72.0)	128,142 (78.6)
Oral prednisolone	61,256 (4.0)	973 (7.7)	7,292 (4.5)
Hydroxychloroquine	16,783 (1.1)	784 (6.2)	4,320 (2.7)
Other DMARDs	48,819 (3.2)	2,016 (16.1)	10,737 (6.6)

Abbreviations: COPD, Chronic obstructive pulmonary disease; DMARDs, Disease-modifying antirheumatic drugs.

Wong AYS, *et al. Ann Rheum Dis* 2021;0:1–9. doi: 10.1136/annrheumdis-2020-219517

Table S9. Descriptive Characteristics in people prescribed NSAIDs in the past 3 years, stratified by ibuprofen

	Non-use of NSAIDs	Current use of Ibuprofen	Current use of other NSAIDs
**Total**	1,927,284	50,688	485,735
**Age as of 1st March 2020**			
18-<40	598,513 (31.1)	6,635 (13.1)	109,223 (22.5)
40-<50	397,201 (20.6)	6,414 (12.7)	96,662 (19.9)
50-<60	423,937 (22.0)	10,324 (20.4)	122,742 (25.3)
60-<70	283,639 (14.7)	12,371 (24.4)	93,834 (19.3)
70-<80	169,281 (8.8)	11,006 (21.7)	51,215 (10.5)
80+	54,713 (2.8)	3,938 (7.8)	12,059 (2.5)
**Sex**			
Female	1,093,581 (56.7)	31,481 (62.1)	285,860 (58.9)
**Body mass index**			
<18.5	26,435 (1.4)	780 (1.5)	5,261 (1.1)
18.5-24.9	484,862 (25.2)	10,869 (21.4)	103,788 (21.4)
25-29.9	577,087 (29.9)	15,149 (29.9)	144,424 (29.7)
30-34.9	333,254 (17.3)	10,284 (20.3)	96,030 (19.8)
35-39.9	138,059 (7.2)	4,959 (9.8)	45,447 (9.4)
40+	71,503 (3.7)	3,006 (5.9)	27,432 (5.6)
Missing	296,084 (15.4)	5,641 (11.1)	63,353 (13.0)
**Ethnicity**			
White	1,236,854 (64.2)	33,752 (66.6)	323,899 (66.7)
Mixed	20,556 (1.1)	364 (0.7)	4,332 (0.9)
Asian or Asian British	151,533 (7.9)	2,950 (5.8)	30,060 (6.2)
Black	49,618 (2.6)	832 (1.6)	9,695 (2.0)
Other	30,214 (1.6)	625 (1.2)	6,300 (1.3)
Missing	438,509 (22.8)	12,165 (24.0)	111,449 (22.9)
**Index of Multiple Deprivation**			
1 (least deprived)	388,369 (20.2)	9,509 (18.8)	98,032 (20.2)
2	387,428 (20.1)	9,868 (19.5)	99,129 (20.4)
3	382,357 (19.8)	10,606 (20.9)	97,020 (20.0)
4	384,598 (20.0)	10,062 (19.9)	96,536 (19.9)
5 (most deprived)	384,532 (20.0)	10,643 (21.0)	95,018 (19.6)
**Smoking status**			
Never	841,256 (43.6)	20,449 (40.3)	199,844 (41.1)
Former	665,068 (34.5)	20,672 (40.8)	186,682 (38.4)
Current	389,340 (20.2)	9,115 (18.0)	94,143 (19.4)
Missing	31,620 (1.6)	452 (0.9)	5,066 (1.0)
**Comorbidities**			
Hypertension	353,803 (18.4)	16,184 (31.9)	111,894 (23.0)
Heart Failure	9,512 (0.5)	388 (0.8)	2,045 (0.4)
Other Heart Disease	27,881 (1.4)	1,104 (2.2)	7,622 (1.6)
Diabetes			
Controlled (HbA1c <	122,653 (6.4)	5,371 (10.6)	36,761 (7.6)

Wong AYS, *et al. Ann Rheum Dis* 2021;0:1–9. doi: 10.1136/annrheumdis-2020-219517

58mmols/mol)			
Uncontrolled (HbA1c ≥ 58mmols/mol)	50,268 (2.6)	2,235 (4.4)	14,269 (2.9)
HbA1c not measured	4,536 (0.2)	138 (0.3)	1,165 (0.2)
COPD	42,636 (2.2)	2,316 (4.6)	13,119 (2.7)
Other respiratory diseases	17,270 (0.9)	1,028 (2.0)	5,166 (1.1)
Cancer	95,315 (4.9)	5,454 (10.8)	26,674 (5.5)
Immunosuppression	9,285 (0.5)	416 (0.8)	2,502 (0.5)
Chronic kidney disease	51,642 (2.7)	2,718 (5.4)	14,852 (3.1)
Osteoarthritis	367,954 (19.1)	20,614 (40.7)	142,062 (29.2)
Rheumatoid arthritis	28,690 (1.5)	2,185 (4.3)	19,341 (4.0)
**GP consultations**			
Median, IQR	5 (2-10)	8 (4-13)	7 (4-13)
Min, Max	0, 626	0, 576	0, 565
**A&E attendance**			
Median, IQR	0 (0-0)	0 (0-0)	0 (0-0)
Min, Max	0, 118	0, 67	0, 152
**Vaccination**			
Flu	435,383 (22.6)	23,755 (46.9)	138,327 (28.5)
Pneumococcal	116,464 (6.0)	5,924 (11.7)	38,978 (8.0)
**Medications**			
Statin	223,221 (11.6)	12,176 (24.0)	74,993 (15.4)
Proton pump inhibitors	268,934 (14.0)	32,965 (65.0)	309,301 (63.7)
Oral prednisolone	39,081 (2.0)	1,934 (3.8)	14,150 (2.9)
Hydroxychloroquine	8,074 (0.4)	613 (1.2)	6,067 (1.2)
Other DMARDs	20,770 (1.1)	1,565 (3.1)	15,292 (3.1)

Abbreviations: COPD, Chronic obstructive pulmonary disease; DMARDs, Disease-modifying antirheumatic drugs.

Wong AYS, *et al. Ann Rheum Dis* 2021;0:1–9. doi: 10.1136/annrheumdis-2020-219517

Table S10. Descriptive Characteristics in people with osteoarthritis/ rheumatoid arthritis, stratified by ibuprofen

	Non-use of NSAIDs	Current use of Ibuprofen	Current use of other NSAIDs
**Total**	1,533,286	21,877	153,618
**Age as of 1st March 2020**			
18-<40	32,958 (2.1)	318 (1.5)	4,115 (2.7)
40-<50	97,870 (6.4)	1,096 (5.0)	14,717 (9.6)
50-<60	292,186 (19.1)	3,687 (16.9)	41,710 (27.2)
60-<70	416,489 (27.2)	6,744 (30.8)	50,203 (32.7)
70-<80	436,477 (28.5)	7,234 (33.1)	34,116 (22.2)
80+	257,306 (16.8)	2,798 (12.8)	8,757 (5.7)
**Sex**			
Female	951,417 (62.1)	14,420 (65.9)	96,106 (62.6)
**Body mass index**			
<18.5	19,616 (1.3)	232 (1.1)	1,028 (0.7)
18.5-24.9	379,233 (24.7)	4,288 (19.6)	27,243 (17.7)
25-29.9	518,602 (33.8)	6,990 (32.0)	48,397 (31.5)
30-34.9	298,505 (19.5)	4,875 (22.3)	35,638 (23.2)
35-39.9	119,286 (7.8)	2,331 (10.7)	17,731 (11.5)
40+	58,801 (3.8)	1,400 (6.4)	10,996 (7.2)
Missing	139,243 (9.1)	1,761 (8.0)	12,585 (8.2)
**Ethnicity**			
White	1,095,982 (71.5)	15,490 (70.8)	109,583 (71.3)
Mixed	6,563 (0.4)	86 (0.4)	744 (0.5)
Asian or Asian British	51,587 (3.4)	869 (4.0)	6,100 (4.0)
Black	17,645 (1.2)	182 (0.8)	1,924 (1.3)
Other	10,916 (0.7)	144 (0.7)	1,097 (0.7)
Missing	350,593 (22.9)	5,106 (23.3)	34,170 (22.2)
**Index of Multiple Deprivation**			
1 (least deprived)	313,701 (20.5)	3,806 (17.4)	26,991 (17.6)
2	309,372 (20.2)	4,023 (18.4)	28,923 (18.8)
3	307,669 (20.1)	4,474 (20.5)	30,123 (19.6)
4	303,859 (19.8)	4,649 (21.3)	32,033 (20.9)
5 (most deprived)	298,685 (19.5)	4,925 (22.5)	35,548 (23.1)
**Smoking status**			
Never	672,833 (43.9)	8,708 (39.8)	61,575 (40.1)
Former	692,164 (45.1)	10,346 (47.3)	70,637 (46.0)
Current	164,464 (10.7)	2,787 (12.7)	21,126 (13.8)
Missing	3,825 (0.2)	36 (0.2)	280 (0.2)
**Comorbidities**			
Hypertension	625,247 (40.8)	9,193 (42.0)	56,905 (37.0)
Heart Failure	36,888 (2.4)	249 (1.1)	1,164 (0.8)
Other Heart Disease	57,976 (3.8)	607 (2.8)	3,595 (2.3)
Diabetes			
Controlled (HbA1c <	177,397 (11.6)	2,712 (12.4)	16,823 (11.0)

Wong AYS, *et al. Ann Rheum Dis* 2021;0:1–9. doi: 10.1136/annrheumdis-2020-219517

58mmols/mol)			
Uncontrolled (HbA1c ≥ 58mmols/mol)	58,452 (3.8)	920 (4.2)	5,366 (3.5)
HbA1c not measured	3,695 (0.2)	48 (0.2)	371 (0.2)
COPD	85,858 (5.6)	1,297 (5.9)	7,076 (4.6)
Other respiratory diseases	38,248 (2.5)	557 (2.5)	2,878 (1.9)
Cancer	174,647 (11.4)	2,748 (12.6)	13,192 (8.6)
Immunosuppression	8,498 (0.6)	133 (0.6)	876 (0.6)
Chronic kidney disease	164,985 (10.8)	1,753 (8.0)	9,395 (6.1)
Osteoarthritis	1,473,833 (96.1)	20,614 (94.2)	142,062 (92.5)
Rheumatoid arthritis	95,056 (6.2)	2,185 (10.0)	19,341 (12.6)
**GP consultations**			
Median, IQR	6 (3-11)	8 (5-14)	8 (5-14)
Min, Max	0, 468	0, 260	0, 360
**A&E attendance**			
Median, IQR	0 (0-0)	0 (0-0)	0 (0-0)
Min, Max	0, 71	0, 63	0, 36
**Vaccination**			
Flu	806,064 (52.6)	13,116 (60.0)	73,399 (47.8)
Pneumococcal	193,145 (12.6)	3,337 (15.3)	21,407 (13.9)
**Medications**			
Statin	415,120 (27.1)	6,860 (31.4)	40,160 (26.1)
Proton pump inhibitors	371,464 (24.2)	16,363 (74.8)	120,817 (78.6)
Oral prednisolone	61,256 (4.0)	1,105 (5.1)	7,160 (4.7)
Hydroxychloroquine	16,783 (1.1)	474 (2.2)	4,630 (3.0)
Other DMARDs	48,819 (3.2)	1,221 (5.6)	11,532 (7.5)

Abbreviations: COPD, Chronic obstructive pulmonary disease; DMARDs, Disease-modifying antirheumatic drugs.

Wong AYS, *et al. Ann Rheum Dis* 2021;0:1–9. doi: 10.1136/annrheumdis-2020-219517

Figure S10. Time to COVID-19 related death by types of NSAIDs in Kaplan-Meier plot – General population

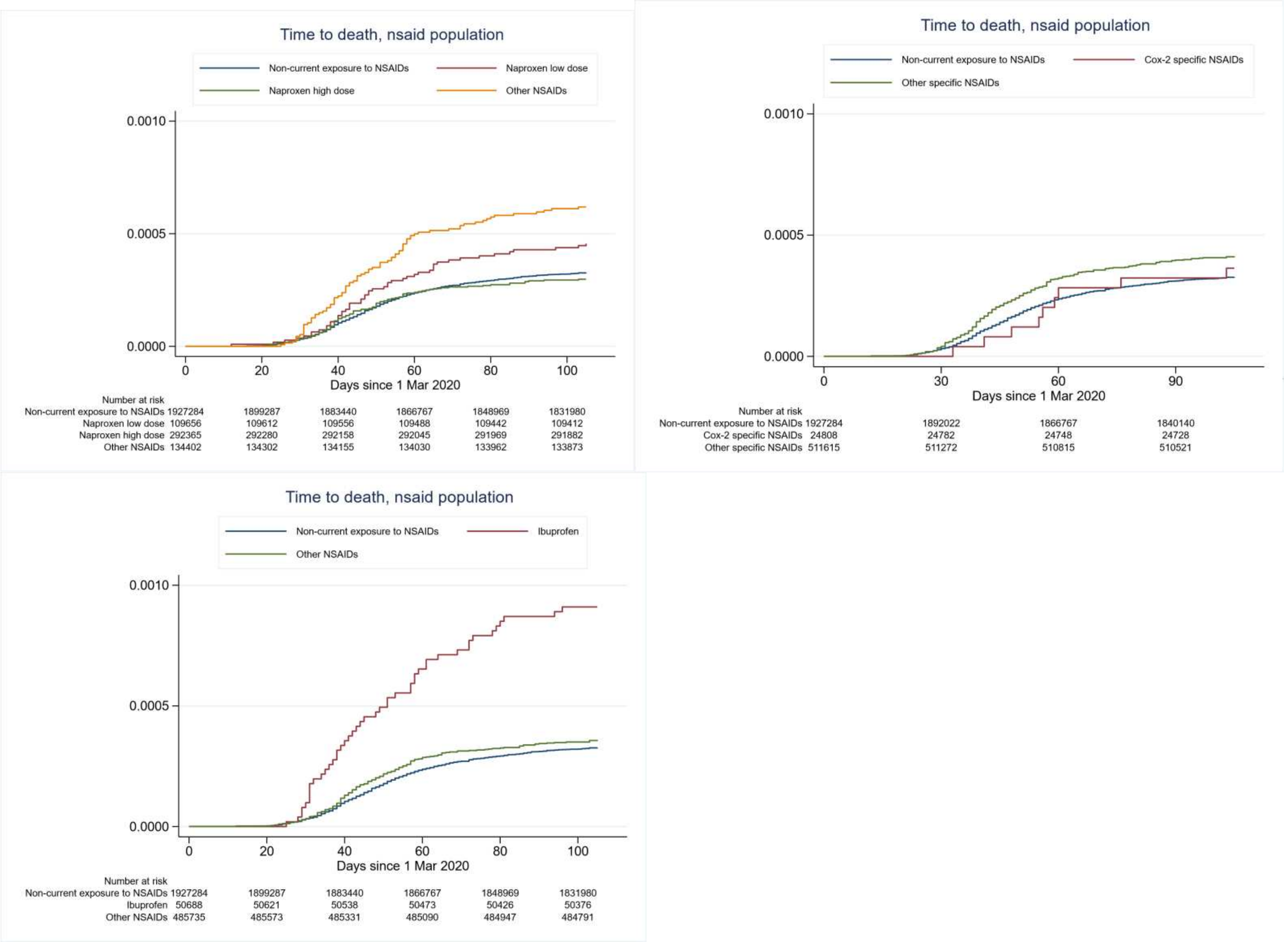

Figure S11. Time to COVID-19 related death by types of NSAIDs in Kaplan-Meier plot – osteoarthritis/ rheumatoid arthritis population

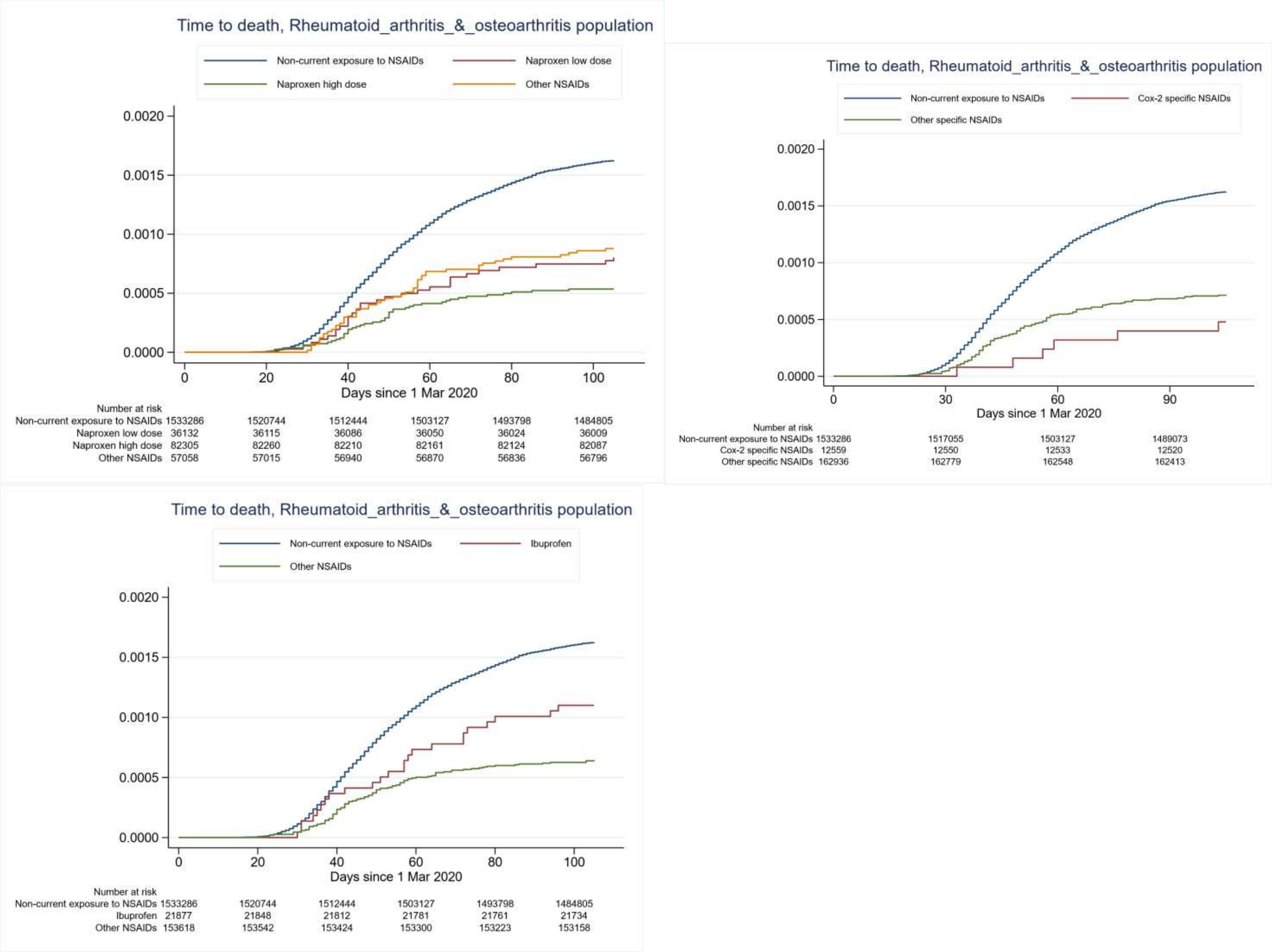

Table S11. Association between low dose naproxen, high dose naproxen and other NSAIDs and COVID-19 compared with non-use of NSAIDs

	Number of deaths	Total person-week	Rate per 1000	Unadjusted		Age/Sex Adjusted		Multivariable-adjusted[a]	
				HR	95% CI	HR	95% CI	HR	95% CI
**Study population 1: General population (NSAIDs prescribed cohort)**									
**Non-use of NSAID**	612	28,098,800	0.02	1.00 (ref)		1.00 (ref)		1.00 (ref)	
**Low dose naproxen**	50	1,642,787	0.03	1.4	1.05 - 1.87	1.08	0.81 - 1.44	0.93	0.69 - 1.25
**High dose naproxen**	87	4,381,525	0.02	0.91	0.73 - 1.14	1.05	0.84 - 1.32	0.92	0.72 - 1.17
**Other NSAIDs**	83	2,011,570	0.04	1.9	1.51 - 2.38	1.15	0.91 - 1.44	1.01	0.80 - 1.29
**Study population 2: Rheumatoid arthritis/ osteoarthritis cohort**									
**Non-use of NSAID**	2450	22,598,389	0.11	1.00 (ref)		1.00 (ref)		1.00 (ref)	
**Low dose naproxen**	29	540,985	0.05	0.49	0.34 - 0.71	0.83	0.58 - 1.20	0.77	0.53 - 1.11
**High dose naproxen**	44	1,232,759	0.04	0.33	0.24 - 0.44	0.85	0.63 - 1.15	0.79	0.58 - 1.07
**Other NSAIDs**	50	853,672	0.06	0.54	0.41 - 0.71	0.81	0.62 - 1.08	0.77	0.58 - 1.02

[a]Adjusted for age, sex, obesity, smoking status, deprivation, chronic kidney disease, hypertension, heart failure, other heart diseases, diabetes severity, chronic obstructive pulmonary disease, other respiratory diseases, immunosuppression, cancer, rheumatoid arthritis (study population 1), osteoarthritis (study population 1), rheumatoid arthritis/osteoarthritis/both (study population 2), statins, proton pump inhibitors, oral prednisolone, hydroxychloroquine, other DMARDS, flu vaccine and pneumococcal vaccine.

Table S12. Association between COX-2 specific NSAIDs, non-specific NSAIDs with COVID-19 death compared with non-use of NSAIDs

	Number of deaths	Total person-week	Rate per 1000	Unadjusted		Age/Sex Adjusted		Multivariable-adjusted[a]	
				HR	95% CI	HR	95% CI	HR	95% CI
**Study population 1: General population (NSAIDs prescribed cohort)**									
**Non-use of NSAID**	612	28,098,800	0.02	1.00 (ref)		1.00 (ref)		1.00 (ref)	
**COX-2 specific NSAIDs**	9	371,411	0.02	1.11	0.58 - 2.15	0.77	0.40 - 1.48	0.61	0.31 - 1.18
**Non-specific NSAIDs**	211	7,664,471	0.03	1.26	1.08 - 1.48	1.11	0.95 - 1.30	0.98	0.82 - 1.17
**Study population 2: Rheumatoid arthritis/ osteoarthritis cohort**									
**Non-use of NSAID**	2450	22,598,389	0.11	1.00 (ref)		1.00 (ref)		1.00 (ref)	
**COX-2 specific NSAIDs**	6	188,073	0.03	0.29	0.13 - 0.66	0.56	0.25 - 1.26	0.48	0.22 - 1.08
**Non-specific NSAIDs**	117	2,439,343	0.05	0.44	0.37 - 0.53	0.85	0.71 - 1.03	0.80	0.66 - 0.97

[a]Adjusted for age, sex, obesity, smoking status, deprivation, chronic kidney disease, hypertension, heart failure, other heart diseases, diabetes severity, chronic obstructive pulmonary disease, other respiratory diseases, immunosuppression, cancer, rheumatoid arthritis (study population 1), osteoarthritis (study population 1), rheumatoid arthritis/osteoarthritis/both (study population 2), statins, proton pump inhibitors, oral prednisolone, hydroxychloroquine, other DMARDS, flu vaccine and pneumococcal vaccine.

Table S13. Association between ibuprofen, other NSAIDs with COVID-19 death compared with non-use of NSAIDs

	Number of deaths	Total person-week	Rate per 1000	Unadjusted		Age/Sex Adjusted		Multivariable-adjusted[a]	
				HR	95% CI	HR	95% CI	HR	95% CI
**Study population 1: General population (NSAIDs prescribed cohort)**									
**Non-use of NSAID**	612	28,098,800	0.02	1.00 (ref)		1.00 (ref)		1.00 (ref)	
**Ibuprofen**	46	757,664	0.06	2.79	2.07 - 3.76	1.35	1.00 - 1.82	1.23	0.90 - 1.68
**Other NSAIDs**	174	7,278,218	0.02	1.10	0.93 - 1.30	1.04	0.88 - 1.23	0.90	0.75 - 1.08
**Study population 2: Rheumatoid arthritis/ osteoarthritis cohort**									
**Non-use of NSAID**	2450	22,598,389	0.11	1.00 (ref)		1.00 (ref)		1.00 (ref)	
**Ibuprofen**	24	326,968	0.07	0.68	0.45 - 1.01	0.85	0.57 - 1.27	0.83	0.56 - 1.25
**Other NSAIDs**	99	2,300,448	0.04	0.40	0.32 - 0.49	0.83	0.68 - 1.02	0.76	0.62 - 0.94

[a]Adjusted for age, sex, obesity, smoking status, deprivation, chronic kidney disease, hypertension, heart failure, other heart diseases, diabetes severity, chronic obstructive pulmonary disease, other respiratory diseases, immunosuppression, cancer, rheumatoid arthritis (study population 1), osteoarthritis (study population 1), rheumatoid arthritis/osteoarthritis/both (study population 2), statins, proton pump inhibitors, oral prednisolone, hydroxychloroquine, other DMARDS, flu vaccine and pneumococcal vaccine.

Table S14. Sensitivity analysis that excluding people ever prescribed aspirin

	Number of deaths	Total person-week	Rate per 1000	Unadjusted		Age/Sex Adjusted HR		Multivariable-adjusted[a]	
				HR	95% CI	HR	95% CI	HR	95% CI
**Study population 1: General population (NSAIDs prescribed cohort)**									
**Non-use of NSAID**	564	27,457,627	0.02	1.00 (ref)		1.00 (ref)		1.00 (ref)	
**Current use of NSAIDs**	209	7,807,712	0.03	1.30	1.11 - 1.53	1.12	0.96 - 1.31	0.98	0.82 - 1.17
**Study population 2: Rheumatoid arthritis/ osteoarthritis cohort**									
**Non-use of NSAID**	2071	21,136,466	0.10	1.00 (ref)		1.00 (ref)		1.00 (ref)	
**Current use of NSAIDs**	117	2,502,595	0.05	0.48	0.40 - 0.57	0.90	0.74 - 1.09	0.84	0.69 - 1.02

[a]Adjusted for age, sex, obesity, smoking status, deprivation, chronic kidney disease, hypertension, heart failure, other heart diseases, diabetes severity, chronic obstructive pulmonary disease, other respiratory diseases, immunosuppression, cancer, rheumatoid arthritis (study population 1), osteoarthritis (study population 1), rheumatoid arthritis/osteoarthritis/both (study population 2), statins, proton pump inhibitors, oral prednisolone, hydroxychloroquine, other DMARDS, flu vaccine, and pneumococcal vaccine.

Wong AYS, *et al. Ann Rheum Dis* 2021;0:1–9. doi: 10.1136/annrheumdis-2020-219517

Table S15. *Post-hoc* analysis that using Directed Acyclic Graph approach to select covariates

	Full cohort					Complete case cohort						
	Number of deaths	Total person-week	Rate per 1000	Adjusted[a]		Number of deaths	Total person-week	Rate per 1000	Adjusted[b]		Adjusted[c]	
				HR	95% CI				HR	95% CI	HR	95% CI
**Study population 1: General population (NSAIDs prescribed cohort)**												
**Non-use of NSAID**	612	28,098,800	0.02	1.00 (ref)		464	21,700,642	0.02	1.00 (ref)		1.00 (ref)	
**Current use of NSAIDs**	220	8,035,882	0.03	1.13	0.96 - 1.32	167	6,184,511	0.03	1.13	0.95 - 1.35	1.14	0.95 - 1.36
**Study population 2: Rheumatoid arthritis/ osteoarthritis cohort**												
**Non-use of NSAID**	2450	22,598,389	0.11	1.00 (ref)		1817	17,430,871	0.10	1.00 (ref)		1.00 (ref)	
**Current use of NSAIDs**	123	2,627,417	0.05	0.86	0.71 - 1.03	86	2039,674	0.04	0.80	0.64 - 0.99	0.79	0.64 - 0.99

[a]Included all covariates selected using DAG approach except ethnicity in full cohort. Adjusted for age, sex, obesity, smoking status, deprivation, chronic kidney disease, hypertension, heart failure, other heart diseases, diabetes severity, chronic obstructive pulmonary disease, other respiratory diseases, immunosuppression, cancer, rheumatoid arthritis (study population 1), osteoarthritis (study population 1), rheumatoid arthritis/osteoarthritis/both (study population 2), oral prednisolone, hydroxychloroquine, other DMARDS, and A&E attendance.

[b]Included all covariates selected using DAG approach except ethnicity in complete case cohort. Adjusted for age, sex, obesity, smoking status, deprivation, chronic kidney disease, hypertension, heart failure, other heart diseases, diabetes severity, chronic obstructive pulmonary disease, other respiratory diseases, immunosuppression, cancer, rheumatoid arthritis (study population 1), osteoarthritis (study population 1), rheumatoid arthritis/osteoarthritis/both (study population 2), oral prednisolone, hydroxychloroquine, other DMARDS, ethnicity and A&E attendance.

[c]Included all covariates selected using DAG approach in complete case cohort. Adjusted for age, sex, obesity, smoking status, deprivation, chronic kidney disease, hypertension, heart failure, other heart diseases, diabetes severity, chronic obstructive pulmonary disease, other respiratory diseases, immunosuppression, cancer, rheumatoid arthritis (study population 1), osteoarthritis (study population 1), rheumatoid arthritis/osteoarthritis/both (study population 2), oral prednisolone, hydroxychloroquine, other DMARDS, ethnicity and A&E attendance.

Table S16. Sensitivity analysis that additionally adjusting for ethnicity in the Multivariable-adjusted models

	Number of deaths	Total person-week	Rate per 1000	Unadjusted		Age/Sex Adjusted		Multivariable-adjusted[a]	
				HR	95% CI	HR	95% CI	HR	95% CI
**Study population 1: General population (NSAIDs prescribed cohort)**									
**Non-use of NSAID**	464	2,170,0642	0.02	1.00 (ref)		1.00 (ref)		1.00 (ref)	
**Current use of NSAIDs**	167	6,184,511	0.03	1.26	1.06 - 1.51	1.10	0.92 - 1.31	1.01	0.83 - 1.24
**Study population 2: Rheumatoid arthritis/ osteoarthritis cohort**									
**Non-use of NSAID**	1817	17,430,871	0.1	1.00 (ref)		1.00 (ref)		1.00 (ref)	
**Current use of NSAIDs**	86	2,039,674	0.04	0.40	0.33 - 0.50	0.78	0.63 - 0.97	0.73	0.58 - 0.91

[a]Adjusted for age, sex, obesity, smoking status, deprivation, chronic kidney disease, hypertension, heart failure, other heart diseases, diabetes severity, chronic obstructive pulmonary disease, other respiratory diseases, immunosuppression, cancer, rheumatoid arthritis, osteoarthritis (study population 1), osteoarthritis (study population 1), rheumatoid arthritis/osteoarthritis/both (study population 2), statins, proton pump inhibitors, oral prednisolone, hydroxychloroquine, other DMARDS, flu vaccine, pneumococcal vaccine and ethnicity. Complete case analysis was conducted for addressing missing data.

Wong AYS, *et al. Ann Rheum Dis* 2021;0:1–9. doi: 10.1136/annrheumdis-2020-219517

Table S17. Sensitivity analysis that additionally adjusted for GP consultation and A&E attendance in the Multivariable-adjusted models

	Number of deaths	Total person-week	Rate per 1000	Unadjusted		Age/Sex Adjusted		Multivariable-adjusted[a]	
				HR	95% CI	HR	95% CI	HR	95% CI
**Study population 1: General population (NSAIDs prescribed cohort)**									
**Non-use of NSAID**	612	28,098,800	0.02	1.00 (ref)		1.00 (ref)		1.00 (ref)	
**Current use of NSAIDs**	220	8,035,882	0.03	1.26	1.08 - 1.47	1.09	0.94 - 1.28	0.98	0.83 - 1.17
**Study population 2: Rheumatoid arthritis/ osteoarthritis cohort**									
**Non-use of NSAID**	2450	22,598,389	0.11	1.00 (ref)		1.00 (ref)		1.00 (ref)	
**Current use of NSAIDs**	123	2,627,417	0.05	0.43	0.36 - 0.52	0.83	0.69 - 1.00	0.80	0.67 - 0.97

[a]Adjusted for age, sex, obesity, smoking status, deprivation, chronic kidney disease, hypertension, heart failure, other heart diseases, diabetes severity, chronic obstructive pulmonary disease, other respiratory diseases, immunosuppression, cancer, rheumatoid arthritis, osteoarthritis (study population 1), osteoarthritis (study population 1), rheumatoid arthritis/osteoarthritis/both (study population 2), statins, proton pump inhibitors, oral prednisolone, hydroxychloroquine, other DMARDS, flu vaccine, pneumococcal vaccine, and GP consultations and A&E attendances in the past year.

Wong AYS, *et al. Ann Rheum Dis* 2021;0:1–9. doi: 10.1136/annrheumdis-2020-219517

Table S18. Sensitivity analysis that separating HbA1c no measures category from uncontrolled diabetes category in the Multivariable-adjusted models

	Number of deaths	Total person-week	Rate per 1000	Multivariable-adjusted[a] (Main analysis)		Multivariable-adjusted[b] (separating HbA1c no measures category from uncontrolled diabetes category)	
				HR	95% CI	HR	95% CI
**Study population 1: General population (NSAIDs prescribed cohort)**							
**Non-use of NSAID**	612	28,098,800	0.02	1.00 (ref)		1.00 (ref)	
**Current use of NSAIDs**	220	8,035,882	0.03	0.96	0.80 - 1.14	0.96	0.81 - 1.14
**Study population 2: Rheumatoid arthritis/ osteoarthritis cohort**							
**Non-use of NSAID**	2450	22,598,389	0.11	1.00 (ref)		1.00 (ref)	
**Current use of NSAIDs**	123	2,627,417	0.05	0.78	0.64 - 0.94	0.78	0.64 - 0.94

[a]Adjusted for age, sex, obesity, smoking status, deprivation, chronic kidney disease, hypertension, heart failure, other heart diseases, diabetes severity (three categories: no diabetes, controlled diabetes, uncontrolled diabetes), chronic obstructive pulmonary disease, other respiratory diseases, immunosuppression, cancer, rheumatoid arthritis (study population 1), osteoarthritis (study population 1), rheumatoid arthritis/osteoarthritis/both (study population 2), statins, proton pump inhibitors, oral prednisolone, hydroxychloroquine, other DMARDS, flu vaccine and pneumococcal vaccine.

[b]Adjusted for age, sex, obesity, smoking status, deprivation, chronic kidney disease, hypertension, heart failure, other heart diseases, diabetes severity (four categories: no diabetes, controlled diabetes, uncontrolled diabetes, diabetes without HbA1c measures), chronic obstructive pulmonary disease, other respiratory diseases, immunosuppression, cancer, rheumatoid arthritis (study population 1), osteoarthritis (study population 1), rheumatoid arthritis/osteoarthritis/both (study population 2), statins, proton pump inhibitors, oral prednisolone, hydroxychloroquine, other DMARDS, flu vaccine and pneumococcal vaccine.

Wong AYS, *et al. Ann Rheum Dis* 2021;0:1–9. doi: 10.1136/annrheumdis-2020-219517

Table S19. Sensitivity analysis that using 2 months to be the timeframe to identify exposure status

	Number of deaths	Total person-week	Rate per 1000	Unadjusted		Age/Sex Adjusted		Multivariable-adjusted[a]	
				HR	95% CI	HR	95% CI	HR	95% CI
**Study population 1: General population (NSAIDs prescribed cohort)**									
**Non-use of NSAID**	666	30,694,208	0.02	1.00 (ref)		1.00 (ref)		1.00 (ref)	
**Current use of NSAIDs**	166	5,440,475	0.03	1.41	1.19 - 1.67	1.16	0.98 - 1.37	1.03	0.86 - 1.24
**Study population 2: Rheumatoid arthritis/ osteoarthritis cohort**									
**Non-use of NSAID**	2484	23,264,091	0.11	1.00 (ref)		1.00 (ref)		1.00 (ref)	
**Current use of NSAIDs**	89	1,961,714	0.05	0.42	0.34 - 0.52	0.81	0.66 - 1.01	0.75	0.61 - 0.94

[a]Adjusted for age, sex, obesity, smoking status, deprivation, chronic kidney disease, hypertension, heart failure, other heart diseases, diabetes severity, chronic obstructive pulmonary disease, other respiratory diseases, immunosuppression, cancer, rheumatoid arthritis (study population 1), osteoarthritis (study population 1), rheumatoid arthritis/osteoarthritis/both (study population 2), statins, proton pump inhibitors, oral prednisolone, hydroxychloroquine, other DMARDS, flu vaccine, and pneumococcal vaccine.

Table S20. Sensitivity analysis that removing indomethacin from exposure of interest

	Number of deaths	Total person-week	Rate per 1000	Unadjusted		Age/Sex Adjusted		Multivariable-adjusted[a]	
				HR	95% CI	HR	95% CI	HR	95% CI
**Study population 1: General population (NSAIDs prescribed cohort)**									
**Non-use of NSAID**	612	28,098,800	0.02	1.00 (ref)		1.00 (ref)		1.00 (ref)	
**Current use of NSAIDs**	217	7,958,682	0.03	1.25	1.07 - 1.46	1.09	0.94 - 1.28	0.96	0.81 - 1.14
**Study population 2: Rheumatoid arthritis/ osteoarthritis cohort**									
**Non-use of NSAID**	2450	22,598,389	0.11	1.00 (ref)		1.00 (ref)		1.00 (ref)	
**Current use of NSAIDs**	122	2,604,091	0.05	0.43	0.36 - 0.52	0.83	0.69 - 1.00	0.78	0.65 - 0.94

[a]Adjusted for age, sex, obesity, smoking status, deprivation, chronic kidney disease, hypertension, heart failure, other heart diseases, diabetes severity, chronic obstructive pulmonary disease, other respiratory diseases, immunosuppression, cancer, rheumatoid arthritis (study population 1), osteoarthritis (study population 1), rheumatoid arthritis/osteoarthritis/both (study population 2), statins, proton pump inhibitors, oral prednisolone, hydroxychloroquine, other DMARDS, flu vaccine, and pneumococcal vaccine.

Table S21. Sensitivity analysis that not censoring people at NSAID prescription after study start date in the non-use of NSAID group as an intention-to-treat analysis

	Number of deaths	Total person-week	Rate per 1000	Unadjusted		Age/Sex Adjusted		Multivariable-adjusted[a]	
				HR	95% CI	HR	95% CI	HR	95% CI
**Study population 1: General population (NSAIDs prescribed cohort)**									
**Non-use of NSAID**	625	28,885,285	0.02	1.00 (ref)		1.00 (ref)		1.00 (ref)	
**Current use of NSAIDs**	220	8,035,882	0.03	1.27	1.09 - 1.48	1.10	0.94 - 1.28	0.96	0.81 - 1.14
**Study population 2: Rheumatoid arthritis/ osteoarthritis cohort**									
**Non-use of NSAID**	2464	22,916,713	0.11	1.00 (ref)		1.00 (ref)		1.00 (ref)	
**Current use of NSAIDs**	123	2,627,417	0.05	0.44	0.36 - 0.52	0.83	0.69 - 1.00	0.78	0.64 - 0.94

[a]Adjusted for age, sex, obesity, smoking status, deprivation, chronic kidney disease, hypertension, heart failure, other heart diseases, diabetes severity, chronic obstructive pulmonary disease, other respiratory diseases, immunosuppression, cancer, rheumatoid arthritis (study population 1), osteoarthritis (study population 1), rheumatoid arthritis/osteoarthritis/both (study population 2), statins, proton pump inhibitors, oral prednisolone, hydroxychloroquine, other DMARDS, flu vaccine, and pneumococcal vaccine

Figure S12. E-value for the upper 95% CI and point estimate in people with rheumatoid arthritis/ osteoarthritis

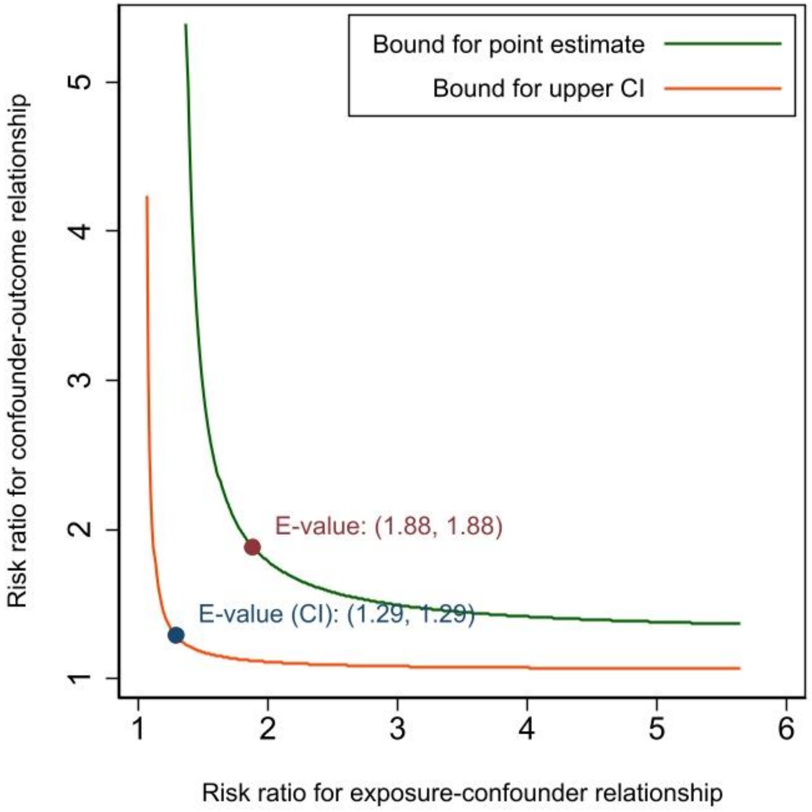

Wong AYS, *et al. Ann Rheum Dis* 2021;0:1–9. doi: 10.1136/annrheumdis-2020-219517
